# Functional diversity of natural human *IFNB1* variants in innate immunity

**DOI:** 10.1126/sciadv.aeh4755

**Published:** 2026-09-18

**Authors:** Samira Schiefer, David Jones, Florence Kwaschik, Benjamin G. Hale

**Affiliations:** Institute of Medical Virology, University of Zurich, 8057 Zurich, Switzerland.

## Abstract

Human interferon-β (IFN-β), a type I IFN, is critical for effective innate immunity and is also an approved disease-modifying therapeutic. While genetic variations in several components of the type I IFN system are associated with infectious and immune diseases, consequences of *IFNB1* variation remain unexplored. Here, we functionally evaluated 70 naturally occurring rare protein-altering *IFNB1* variants detected in humans globally. Twenty-five percent of all protein variants were found to be substantially less active than the common reference IFN-β, a phenotype that for many correlated with retention in the ER and poor secretion, or to compromised interaction with IFNAR1/IFNAR2 cell surface receptors. Notably, three *IFNB1* variants, including one found to be homozygous in a single individual, encode IFN-β proteins with enhanced capacity to engage IFNAR1/IFNAR2 and which exhibit greater signaling and antiviral potency than common IFN-β. Our mechanistic survey of natural human IFN-β functional diversity provides a framework to explore both disease associations and the optimization of human IFN-β in next-generation therapeutics.

## INTRODUCTION

Host innate immunity relies on rapidly activated signaling cascades to respond to pathogens and to up-regulate transcriptional programs that limit the early stages of infection. Interferons (IFNs), a family of cytokines that can coordinate these defenses, are pivotal to this process. Among them, type I IFNs (IFN-Is) are essential for the initiation of antiviral states across virus-infected and neighboring cells. Human IFN-I proteins consist of IFN-β, 12 distinct IFN-αs, IFN-ω, IFN-ε, and IFN-κ (*1*). IFN-β is well characterized as a major antiviral player and can, in contrast to IFN-αs, be produced by most cells, including nonimmune cell types (*1*–*3*).

Upon sensing of virus-derived nucleic acids, cells initiate the phosphorylation and activation of interferon regulatory factor 3 (IRF3) and/or IRF7, the two main transcription factors that trigger production of IFN-Is. IFN-β is predominantly induced by IRF3/7, with nuclear factor κB signaling additionally driving early expression (*1*–*4*). Newly produced IFN-β protein is released from cells via the conventional endoplasmic reticulum (ER) to Golgi secretory pathway (*5*). Secreted IFN-Is can subsequently bind IFN-α/β receptor 1 (IFNAR1) and IFNAR2, which are expressed on the surface of most cell types, and thereby activate the IFN-I signaling cascade. Among IFN-Is, IFN-β has the highest affinity toward both IFNAR1 and IFNAR2 (*2*, *6*, *7*). The IFN-bound IFNAR1/2 heterodimer activates the intracellular kinases, Janus kinase 1, and tyrosine kinase 2 (TYK2), to phosphorylate signal transducer and activator of transcription 1 (STAT1) and STAT2 proteins. Together with IRF9, the phosphorylated STATs act as a transcription factor complex to initiate expression of several hundred distinct IFN-stimulated genes (ISGs), which have diverse but critical antiviral functions that limit viral disease (*1*–*3*).

Given its potent antiviral properties, recombinant IFN-β, like IFN-α2, has been trialed as an antiviral therapy (*2*, *8*), and early administration of IFN-β can enhance survival outcomes during respiratory virus infections (*6*, *9*–*14*). Furthermore, early treatment with IFN-β may have contributed to the recovery of an individual suffering from severe COVID-19 exacerbated by neutralizing autoantibodies against other IFN-Is (*15*). Beyond its antiviral properties, IFN-β has also been used to mitigate multiple sclerosis (MS) disease progression (*2*). In this regard, recombinant bacterially expressed human IFN-β-1b and mammalian-expressed human IFN-β-1a were approved as disease-modifying therapies for MS in the 1990s, followed more recently by the authorization of a pegylated version of IFN-β-1a with enhanced stability (*2*). While IFN-β treatment can delay MS advancement, its longer-term use in individuals is often discontinued due to side effects such as flu-like symptoms or injection site reactions (*16*, *17*). Another drawback of current IFN-β regimens is the potential for development of neutralizing antidrug antibodies, which, at high titers, can render the therapy ineffective (*18*).

Dysregulation of the endogenous IFN-I system can have adverse effects on the host, with consequences including the aggravation of infectious disease progression or autoimmunity (*19*–*21*). Genetic variants in the host are one potential cause of such malfunctions, alongside their immunological phenocopies such as neutralizing anti-IFN-I autoantibodies (*22*, *23*). Hyperactive IFN-I signaling can lead to autoinflammatory disorders, as illustrated by STAT2 gain-of-function variants that are unable to interact with the Ubl carboxyl-terminal hydrolase 18 protein (USP18), a negative regulator of IFN signaling (*24*, *25*). Moreover, several deficiencies in the IFN-I pathway have been associated with enhanced risk for severe infections, including those caused by viruses (*26*). For example, genetic defects in genes encoding different members of the IFN-I signaling cascade, including IFNAR1/2, TYK2, STAT1, and IRF9, as well as in genes encoding IFN-regulated antiviral effectors such as IFITM3 and MX1, have been associated with severe acute respiratory syndrome coronavirus 2 (SARS-CoV-2) or influenza A virus infections (*27*–*33*).

In this study, we aimed to functionally assess the protein products of naturally occurring human protein–altering *IFNB1* genetic variants, with the goal to understand the spectrum of IFN-β activities that may exist in the global population gene pool. We identify multiple IFN-β protein variants that are less active than the common human IFN-β variant and characterize the different molecular and cellular mechanisms that underlie their compromised function. Notably, we also identify three naturally occurring human IFN-β variants that display substantially enhanced IFN signaling activities and antiviral potencies, actions relating to their increased capacities to engage with the IFNAR receptors. Our systematic functional and mechanistic analysis of human IFN-β variants therefore provides a reference point to explore both potential disease associations and the refinement of IFN-β therapies.

## RESULTS

### Functional screening of rare genetic variants in human *IFNB1*

**Fig. 1.**
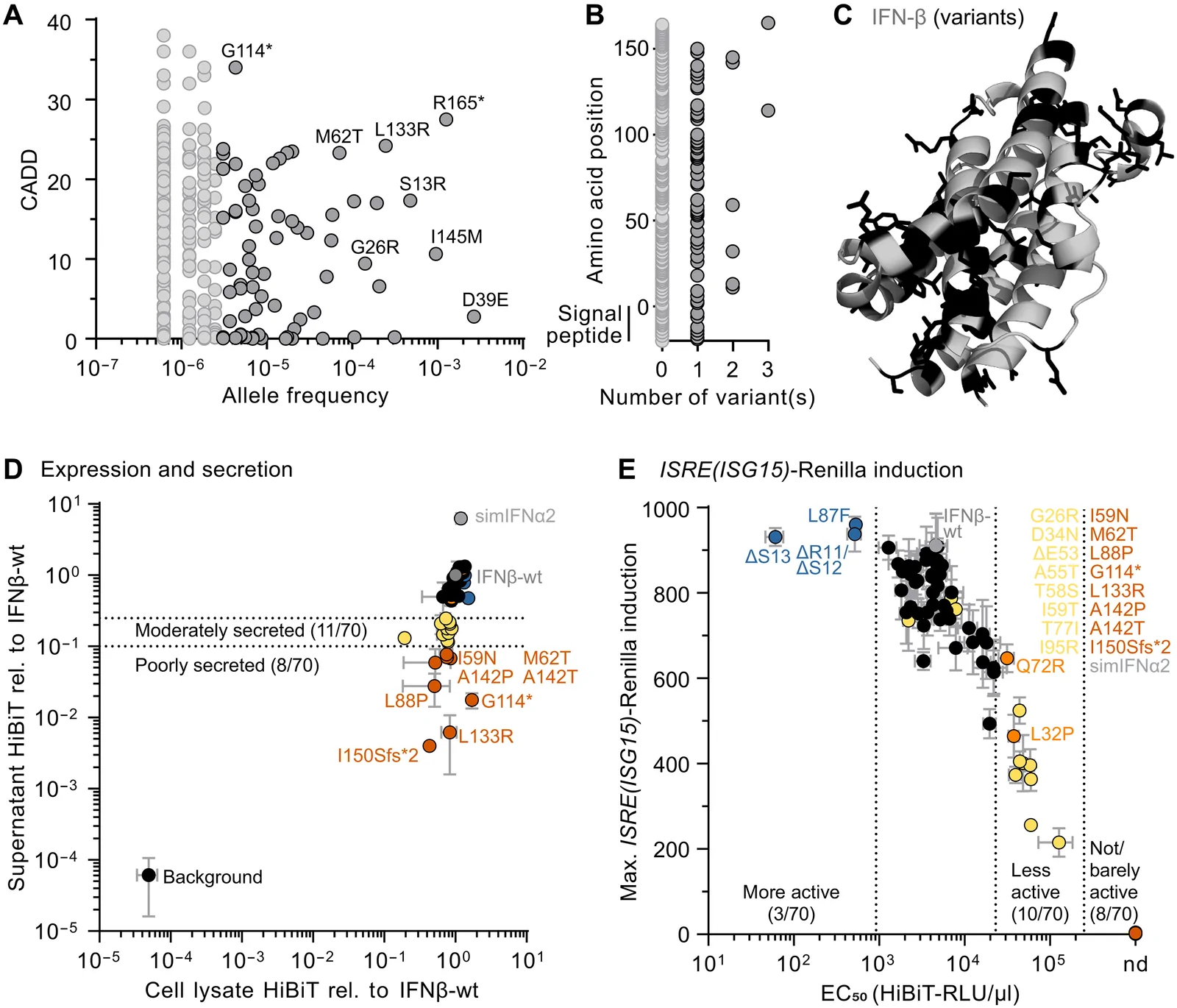
Functional screening of rare genetic variants in human *IFNB1*. (**A**) PHRED-scale CADD (v1.7) scores and AFs of 330 human protein–altering *IFNB1* genetic variants from gnomAD. Dark gray variants were in at least five individuals (AF > 3.1 × 10^−6^); light gray variants were in one to four individuals. See data S1. (**B**) Substituted amino acid positions, referencing mature IFN-β, for the protein-altering rare genetic variants identified in at least five individuals. (**C**) Structure of human IFN-β predicted with ColabFold (*44*, *45*) v1.5.5. Amino acid side chains substituted in the IFN-β variants studied are highlighted in black. (**D**) HEK293T cells were transfected to express HiBiT-tagged IFN-β wild-type (wt), the 70 protein-altering IFN-β variants, or a signaling-inert mutant IFN-α2 [simIFNα2 (*36*); negative control]. HiBiT-tagged protein levels were quantified with the Nano-Glo HiBiT Lytic Detection Assay in supernatant (secretion) and cell lysate (expression) fractions and are plotted relative to IFNβ-wt. Moderately secreted variants (10 to 25% of wt) are highlighted in yellow, while poorly secreted variants (<10% of wt) are highlighted in red. Mean values ± SDs from *n* = 3 independent experiments are shown. (**E**) AIR cells were stimulated for 15 hours with equal quantities (except for poorly secreted variants) of HEK293T supernatant–derived HiBiT-tagged IFN-β (wt or variants), or simIFNα2, in 1:5 serial dilutions. Maximal *ISRE(ISG15)*-Renilla induction over background is plotted against derived effective concentration 50 (EC_50_) values. Moderately secreted variants (10 to 25% of wt) are highlighted in yellow, while poorly secreted variants (<10% of wt) are highlighted in red. More active variants (EC_50_ > fivefold lower than IFNβ-wt) are highlighted in blue, and less active variants (EC_50_ > fivefold greater than IFNβ-wt) that were not impaired in secretion are highlighted in orange. Mean values ± SDs from *n* = 3 independent experiments are shown. See data S1. nd, not determined.

As an unbiased approach to characterizing human *IFNB1* genetic variants, we first interrogated the Genome Aggregation Database (gnomAD v4.1; October 2024), which contains exome and genome data from >700,000 individuals worldwide (*34*). After sequence quality control filtering, 330 human protein–altering *IFNB1* variants (including missense, nonsense, in-frame deletion, and frameshift variants) were identified to exist in at least one individual worldwide, all of which are categorized as rare (<1% prevalence in the general human population; data S1). To permit functional characterization, we focused on variants detected in at least five individuals [allele frequency (AF) > 3 × 10^−6^; Fig. 1A]. This resulted in 71 *IFNB1* genetic variants encoding 70 different IFN-β proteins, including 63 missense variants, 4 in-frame deletions, 2 nonsense variants, and 1 frameshift (data S1). The variants exhibited diverse predictions of deleteriousness [Combined Annotation Dependent Depletion (CADD) score (*35*); Fig. 1A]. For these 71 *IFNB1* genetic variants, substitutions or deletions were observed at 61 distinct residues spread throughout the IFN-β protein, with multiple changes at eight residues (Fig. 1, B and C). We cloned each of the 70 different IFN-β protein-coding variants including their N-terminal signal peptides and were able to express all of them individually in human embryonic kidney (HEK) 293T cells. Each IFN-β variant, as well as the common IFN-β (that we termed IFNβ-wt) and a signaling-inert mutant IFN-α2 [that we termed simIFNα2 (*36*); negative control], were C-terminally tagged with HiBiT. This small tag permitted protein abundance quantification in both cell lysates (for general expression) and supernatants (for secretion) of transfected cells by luminescent HiBiT reporter assay (*37*). All tested variants expressed similarly to IFNβ-wt (Fig. 1D). However, 11 of 70 variants appeared to be moderately impaired in their secretion (12 to 25% of IFNβ-wt in supernatants; Fig. 1D), and another 8 of 70 variants were severely impaired in secretion (0.4 to 10% of IFNβ-wt in supernatants; Fig. 1D).

**Fig. 2.**
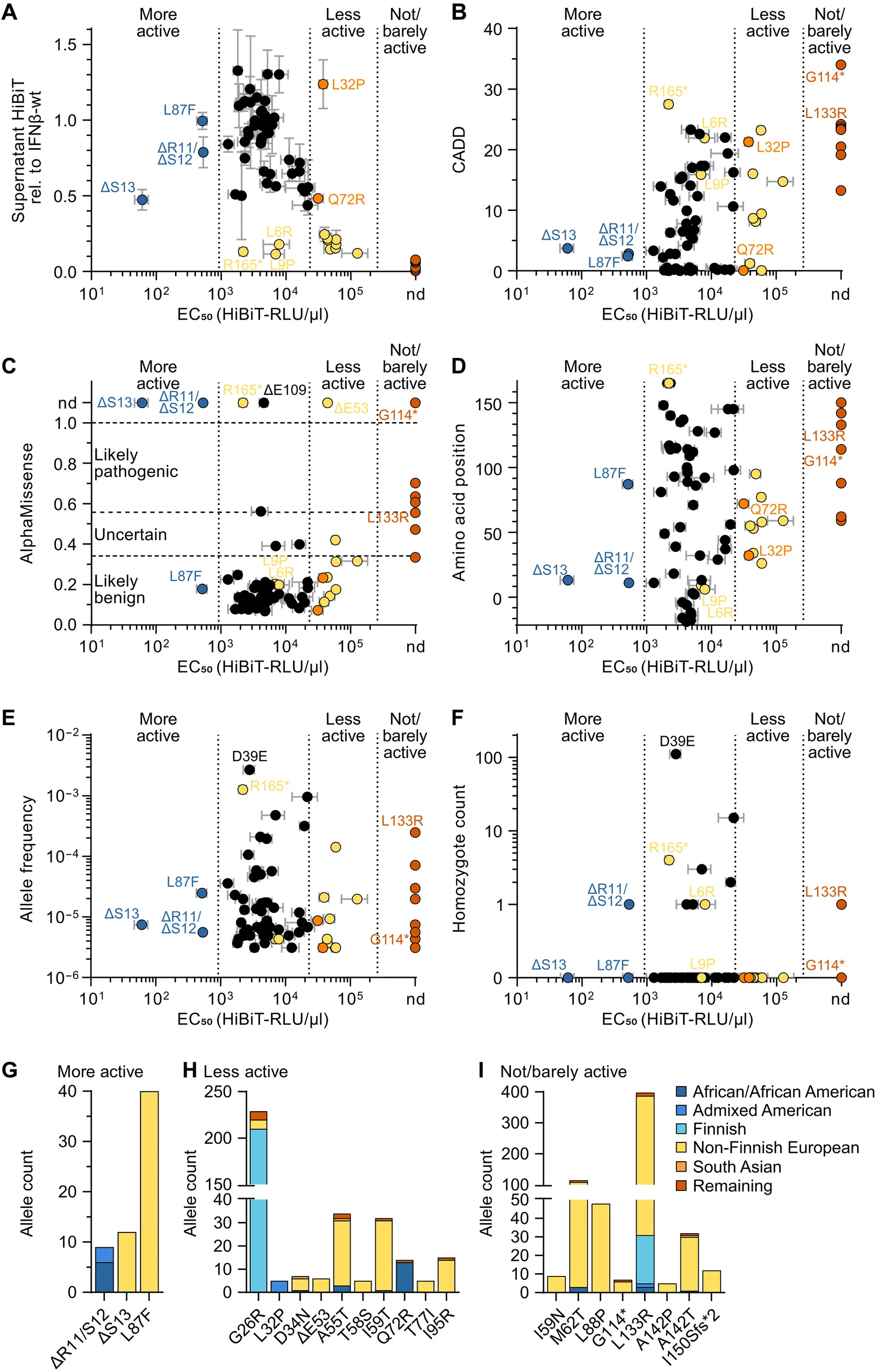
Characterization of rare genetic variants in human *IFNB1*. (**A** to **F**) HEK293T cells were transfected to express HiBiT-tagged IFN-β wt or the 70 protein-altering IFN-β variants. The EC_50_ values of supernatants (also shown in Fig. 1E) are plotted versus the relative HiBiT-tagged protein quantities in the supernatant (also shown in Fig. 1D) (A), versus PHRED-scale CADD [v1.7 (*35*)] scores (B), versus AlphaMissense (*39*) scores (C), versus the amino acid position (D), versus the AF (E), and versus the homozygote count (F) of the relevant IFN-β variants. Moderately secreted variants (10 to 25% of wt) are highlighted in yellow, while poorly secreted variants (<10% of wt) are highlighted in red. More active variants (EC_50_ > fivefold lower than IFNβ-wt) are highlighted in blue, and less active variants (EC_50_ > fivefold greater than IFNβ-wt) that were not impaired in secretion are highlighted in orange. Mean values ± SDs from *n* = 3 independent experiments are shown. (**G** to **I**) The allele counts in different populations for the more active (G), less active (H), or not/barely active (I) protein-altering IFN-β variants identified in Fig. 1E. More detailed information can be found in data S1 and fig. S1.

Next, we sought to compare the signaling activities of the different IFN-β variants. To this end, we made use of a human lung epithelial A549 cell line stably expressing Renilla luciferase under control of an *ISG15* promoter–based IFN-stimulated response element (*ISRE*; AIR cells) (*38*). AIR cells were treated with serial dilutions of HiBiT-normalized quantities of each secreted IFN, and Renilla luciferase activity was determined 15 hours later. For the minimally secreted IFN-β variants, the maximum possible amount of IFN-β was used. Most IFN-β variants (49 of 70) exhibited activities similar to IFNβ-wt in this assay system, with maximal *ISRE*-driven Renilla activities being within a twofold range of IFNβ-wt, and calculated effective concentration 50 (EC_50_) values being within a fivefold range of IFNβ-wt (Fig. 1E). However, 10 of 70 variants were >5 times less active than IFNβ-wt, including G26R, L32P, D34N, ΔE53, A55T, T58S, I59T, Q72R, T77I, and I95R (Fig. 1E). There was no clear correlation between IFN-β variant secretory capacity and activity, as some moderately secreted variants (L6R, L9P, and R165*) maintained activity, while others (L32P and Q72R) were efficiently secreted but exhibited compromised activity (Fig. 2A). No EC_50_ values could be determined for the eight poorly secreted variants (Fig. 1E), indicating that their potential activity at very low concentrations is below the assay detection limit. Notably, 3 of the 70 IFN-β variants (ΔR11/S12, ΔS13, and L87F) were >5 times more active than IFNβ-wt as determined by EC_50_ values (Fig. 1E). Overall, our phenotypic screen of 70 naturally occurring rare human IFN-β variants identified 21 variants with activities that are clearly distinct from the common IFN-β. Functional capacities of these distinct variants could not have been easily predicted in silico, as there was no clear correlation between CADD (Fig. 2B) or AlphaMissense (Fig. 2C) (*39*) scores and empirically tested IFN-β variant activity, although the eight poorly secreted variants all had CADD scores of ≥13 reflecting that they are among the top 5% most likely deleterious variants (Fig. 2B). Moreover, there were no observable correlations between our experimentally determined EC_50_ values and variant amino acid position, AF, or homozygote count (Fig. 2, D to F). Most (17 of 21) variants with activities distinct from common IFN-β were predominantly identified in the non-Finnish European population, which may simply reflect that this group is the most extensively represented within gnomAD-derived *IFNB1* sequences (73% of total *IFNB1* alleles; Fig. 2, G to I, and data S1). However, some IFN-β variants, such as the more active ΔR11/S12 or less active G26R, appeared predominantly in African/African American and Admixed American or Finnish populations, respectively, although the low allele counts prevent the drawing of any definitive conclusions from this observation (Fig. 2, G to I, and fig. S1).

### Poorly secreted IFN-β variants exhibit ER retention

**Fig. 3.**
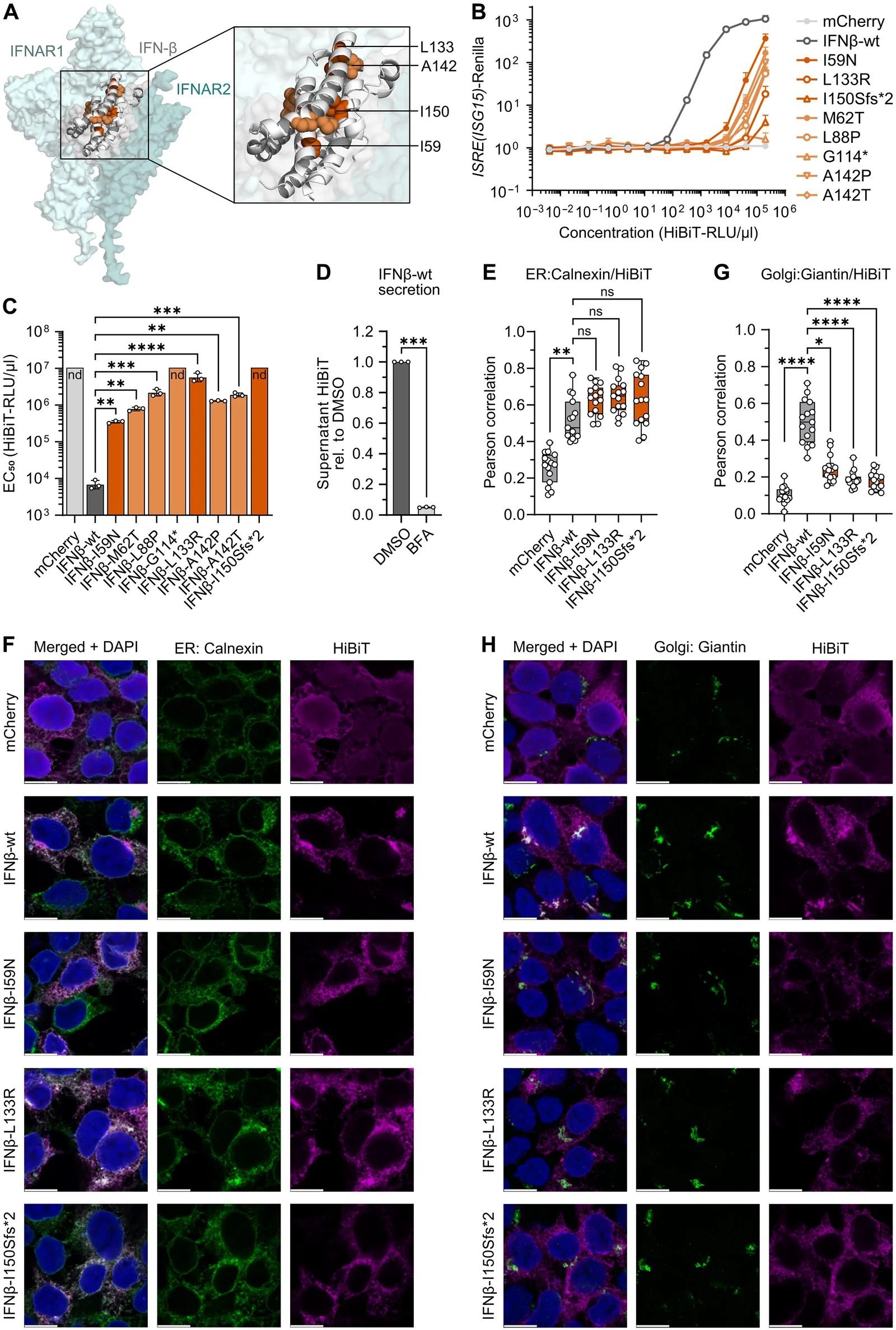
Poorly secreted variants exhibit ER retention. (**A**) Structure of IFN-β with IFNAR1/2 [ColabFold (*44*, *45*) v1.5.5 prediction]. Substituted positions that led to poor secretion are red (I59, L133, and I150) or light red. (**B** and **C**) AIR cells were stimulated for 15 hours with equal quantities of HEK293T cell lysate–derived HiBiT-tagged IFN-β (wt or variants) in a 1:5 dilution series. *ISRE(ISG15)*-Renilla induction versus HiBiT concentration (B), or EC_50_ (C), is plotted. Mean values + SDs (B) or ± SDs (C) from *n* = 3 independent experiments are shown. Significance was determined by one-way analysis of variance (ANOVA) with Dunnett’s multiple comparisons test on the log_10_-transformed values (**P < 0.01, ***P < 0.001, and ****P < 0.0001). Data are also shown in fig. S2A. (**D**) HEK293T cells were transfected with HiBiT-tagged IFNβ-wt and treated with BFA (1 μg/ml) or dimethyl sulfoxide (DMSO) for 6 hours. HiBiT-tagged IFN-β in the supernatant was then quantified. Mean values ± SDs from *n* = 3 independent experiments are shown. Significance was determined by a two-tailed unpaired *t* test with Welch’s correction on the log_10_-transformed values (***P < 0.001). (**E** to **H**) HEK293T cells were transfected with HiBiT-tagged IFN-β (wt or variants) or mCherry. Colocalization (white signal in merge, Pearson correlation coefficient) of ER [calnexin, green; (E) and (F)] or Golgi [giantin, green; (G) and (H)] with HiBiT (magenta) was assessed by immunofluorescence (IF) microscopy. For (E) and (G), single dots represent single images with at least 10 cells. *N* = 5 images were analyzed for *n* = 3 independent experiments (all data shown in plots). Significance was determined by the Kruskal-Wallis test with Dunn’s multiple comparisons (*P < 0.05, **P < 0.01, and ****P < 0.0001; ns, nonsignificant). [(F) and (H)] Representative IF images. Scale bars, 10 μm. Data are also shown in fig. S2 (C and D). DAPI, 4′,6-diamidino-2-phenylindole.

The eight poorly secreted IFN-β variants are among the top 5% predicted most deleterious genomic variants according to their CADD scores (data S1). The corresponding residues are either buried within the tertiary structure of IFN-β (e.g., I59) or encompass potential structurally destabilizing surface substitutions (e.g., A142P; Fig. 3A). As we could not readily stimulate AIR cells with IFNβ-wt equivalent quantities of secreted versions of these eight variants (Fig. 1, D and E), we nevertheless assessed their intrinsic activities by exposing AIR cells to serial dilutions of HiBiT-normalized quantities of cell lysates expressing the variants. Notably, under these conditions, six of the eight poorly secreted IFN-β variants (I59N, M62T, L88P, L133R, A142P, and A142T) were able to induce *ISRE*-Renilla activity more than 10-fold as compared to the mCherry negative control (Fig. 3B and fig. S2A), while the two truncated variants (IFNβ-G114* and I150Sfs*2) showed minimal signaling activities. However, the cell lysate–derived, but poorly secreted, variants were all >50-fold reduced in activity compared to IFNβ-wt as determined by calculated EC_50_ values (Fig. 3C). This effect was not due to destabilization of the IFN-β variants, as Western blotting revealed that variant intracellular levels were typically greater than IFNβ-wt intracellular levels in transfected HEK293T cells, and treatment with the proteasome inhibitor MG132 did not further promote IFN-β variant accumulation (fig. S2B). The increased intracellular level of each IFN-β variant compared to IFNβ-wt is consistent with a secretion defect. These data highlight that the eight IFN-β variants retain only very limited activity and that their overall intrinsic function is severely impaired.

Misfolded IFN-λ4 has been shown to accumulate in the ER and to be poorly secreted (*40*, *41*). We therefore hypothesized that the poorly secreted, and intrinsically defective, IFN-β variants might also be misfolded and exhibit a similar intracellular phenotype. Newly synthesized IFN-β is thought to be normally secreted from cells via a conventional pathway and traffics through the ER to Golgi before release (*5*). We validated that IFNβ-wt is secreted via the conventional secretory pathway in transfected HEK293T cells by confirming that release of HiBiT-tagged IFNβ-wt can be inhibited by brefeldin A (BFA) treatment (Fig. 3D), which blocks Golgi trafficking (*42*). We next sought to understand the mechanism by which well expressed, but poorly secreted, IFN-β variants fail to be released from cells. We therefore expressed three of the eight poorly secreted HiBiT-tagged IFN-β variants (I59N, L133R, and I150Sfs*2; selected as examples due to their distinct substitution locations or properties), in parallel with IFNβ-wt, in HEK293T cells and assessed their subcellular localizations by immunofluorescence microscopy. Transiently transfected cells were costained for the HiBiT tag and the ER marker, calnexin, or the Golgi marker, giantin. The specificity of the immunostaining was confirmed with a nontransfected control (fig. S2C). As compared with the HiBiT-tagged mCherry negative control, a fraction of IFNβ-wt clearly colocalized with the ER marker, and this did not significantly differ for the three poorly secreted variants [I59N, L133R, and I150Sfs*2; white signal in Fig. 3 (E and F)]. As expected, a fraction of HiBiT-tagged IFNβ-wt also specifically colocalized with the Golgi marker [white signal in Fig. 3 (G and H)]. In contrast, colocalization with the Golgi marker was significantly reduced for the three poorly secreted variants when compared to IFNβ-wt [white signal in Fig. 3 (G and H)]. The observation that IFNβ-wt, -I59N, -L133R, and -I150Sfs*2 can all localize to the ER but that only the three poorly secreted variants are significantly less associated with the Golgi (fig. S2D) suggests that these substitutions in IFN-β impair correct protein trafficking from the ER to the Golgi, possibly due to substantial protein misfolding.

### Selected low-potency IFN-β variants exhibit impaired IFNAR1/IFNAR2 receptor engagement, reduced signaling capacity, and diminished antiviral activity

**Fig. 4.**
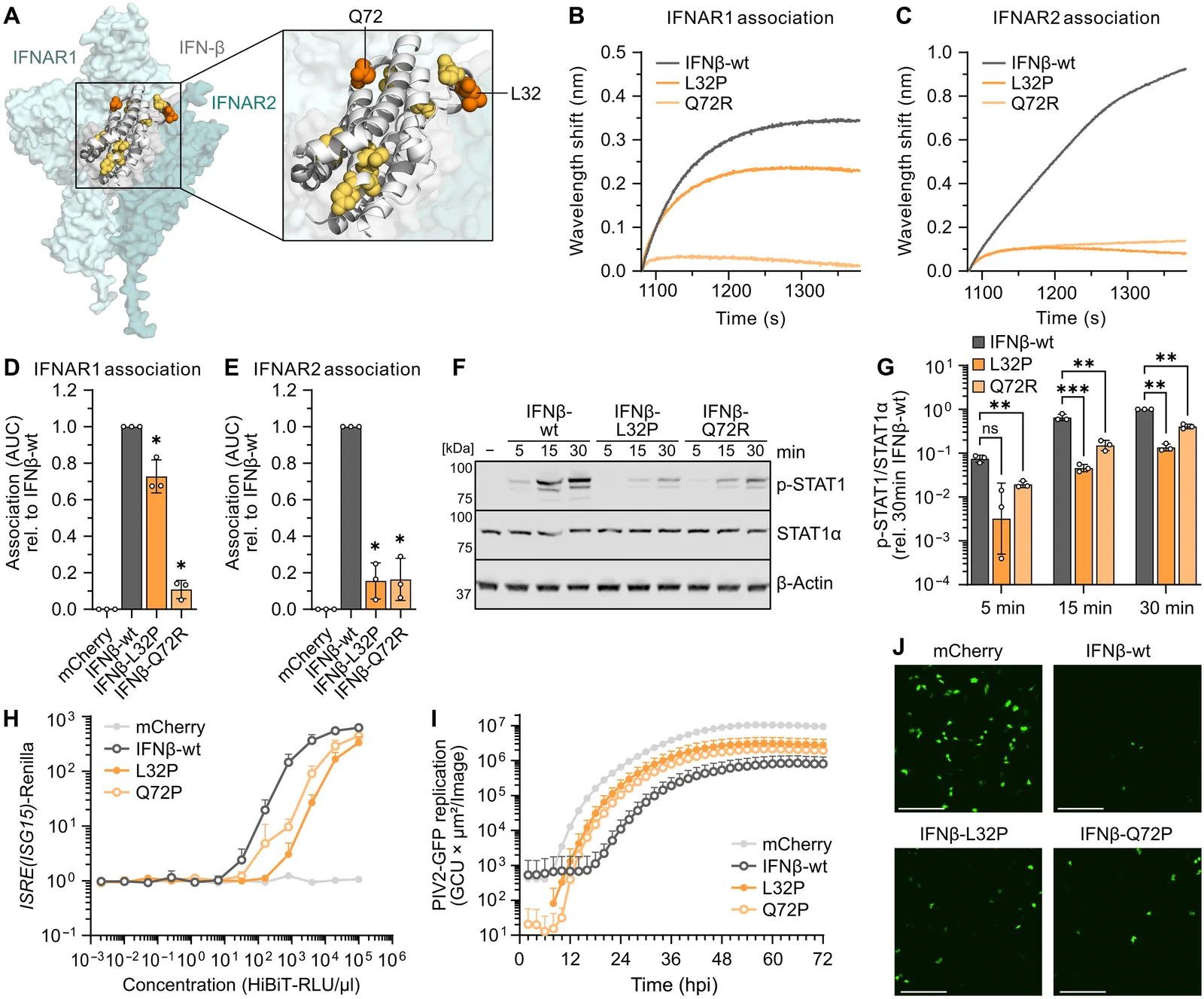
Low-potency IFN-β variants exhibit impaired IFNAR1/2 receptor engagement and diminished antiviral signaling. (**A**) Structure of IFN-β with IFNAR1/2 [ColabFold (*44*, *45*) v1.5.5 prediction]. Substitutions in less active variants are highlighted as orange (L32 and Q72) or yellow. (**B** to **E**) Equal quantities of HiBiT-tagged IFN-β (wt or variants) or mCherry were assessed for binding to IFNAR1/2 with BLI. Average wavelength shifts [(B) and (C)] and the area under the curve (AUC) ± SDs during association [(D) and (E)] from *n* = 3 independent experiments are shown. Individual replicates are shown in fig. S3 (A to F). Significance was determined by a one-sample *t* test toward 0 on the log_10_-transformed values (*P < 0.05). (**F**) A549 cells were stimulated with 2 × 10^5^ HiBiT-RLU/μl of HiBiT-tagged IFN-β (wt or variants) for up to 30 min, and indicated protein levels were determined by Western blot. (**G**) Quantification of STAT1 phosphorylation in (F) from *n* = 3 independent experiments. Mean values ± SDs are shown. Significance was determined by two-way ANOVA with Dunnett’s multiple comparisons test on the log_10_-transformed values (**P < 0.01 and ***P < 0.001). (**H**) AIR cells were stimulated for 6 hours with equal quantities of HiBiT-tagged IFN-β (wt or variants), or mCherry, in a 1:5 dilution series. Mean values + SDs from *n* = 3 independent experiments are shown. (**I**) A549 cells were stimulated with equal quantities of HiBiT-tagged IFN-β (wt or variants), or mCherry, for 6 hours before infection with PIV2-GFP (MOI = 0.1 PFU per cell). Cells were imaged with the IncuCyte system. Mean values + SDs from *n* = 3 independent experiments are shown. Data are also shown in Fig. 5I, as they derive from the same experiments. (**J**) Representative images from the experiment shown in (I) at 24 hours postinfection (hpi). Scale bars, 400 μm.

Of the 10 IFN-β variants that were >5 times less active than IFNβ-wt, 8 variants were also moderately impaired in their secretion capacity (G26R, D34N, ΔE53, A55T, T58S, I59T, T77I, and I95R; 10 to 25% of IFNβ-wt; Fig. 1, D and E). To characterize additional mechanisms that compromise the function of naturally occurring IFN-β variants, we therefore focused on the two low-potency IFN-β variants, with surface-exposed L32P and Q72R substitutions, that had similar secretory profiles to IFNβ-wt (Fig. 4A). Given that the IFN-I signaling cascade is initiated when IFN-β binds to its receptors, IFNAR1 and IFNAR2, we evaluated the relative receptor-binding capacity of the IFN-β variants using biolayer interferometry (BLI). His-tagged recombinant IFNAR1 or IFNAR2 was independently bound to nickel-coated sensor probes and used to measure the in vitro interaction with each secreted HiBiT-tagged IFN-β variant produced by HEK293T cells. Compared with IFNβ-wt, IFNβ-L32P showed a moderate reduction in binding to IFNAR1 but was strongly impaired in its interaction with IFNAR2, consistent with the location of L32 at the interface of IFN-β with IFNAR2 (Fig. 4, A to E, and fig. S3, A to F). In contrast, IFNβ-Q72R was strongly compromised for binding to both IFNAR1 and IFNAR2 (Fig. 4, A to E, and fig. S3, A to F). Western blotting confirmed that similar levels of full-length IFNβ-L32P and IFNβ-wt proteins were used in the assays (fig. S3, G and H). However, Western blotting also revealed that IFNβ-Q72R was slightly reduced in abundance compared to IFNβ-wt (fig. S3, G to I). Nevertheless, a validated excess of IFNβ-Q72R was still weakened in its interaction with IFNAR1/IFNAR2 (fig. S3, I to M). These data indicate that the L32P and Q72R substitutions likely affect receptor interactions through either disruption of specific contacts or localized structural rearrangements in IFN-β, given that any potential variant misfolding is not to a level that limits their secretion (Fig. 1D).

In line with impaired engagement with IFNAR1/2, both IFNβ-L32P and -Q72R variants resulted in markedly reduced STAT1 phosphorylation, as compared with IFNβ-wt, when human lung epithelial A549 cells were stimulated for 5 to 30 min with 2 × 10^5^ HiBiT-RLU/μl of each HiBiT-tagged IFN-β (Fig. 4, F and G). Consequently, *ISRE*-Renilla induction was substantially diminished, as compared with IFNβ-wt, when AIR cells were stimulated for 6 hours with either IFNβ-L32P or -Q72R variants (Fig. 4H). Furthermore, while pretreatment of A549 cells for 6 hours with 1 × 10^5^ HiBiT-RLU/μl of IFNβ-wt was highly protective against subsequent infection with green fluorescent protein–expressing parainfluenza virus type 2 [PIV2-GFP; multiplicity of infection (MOI) = 0.1 plaque-forming units (PFU) per cell], a model human respiratory virus, the same HiBiT quantities of IFNβ-L32P or -Q72R were significantly less antiviral (Fig. 4, I and J, and fig. S3N). These data suggest impaired IFNAR1/IFNAR2 receptor engagement as the mechanistic basis for the compromised function of low-potency IFNβ-L32P and -Q72R variants and reveal the consequences for intracellular signaling and antiviral activity.

### Highly potent IFN-β variants exhibit enhanced IFNAR1/IFNAR2 receptor engagement, altered signaling magnitudes, and greater antiviral activities

**Fig. 5.**
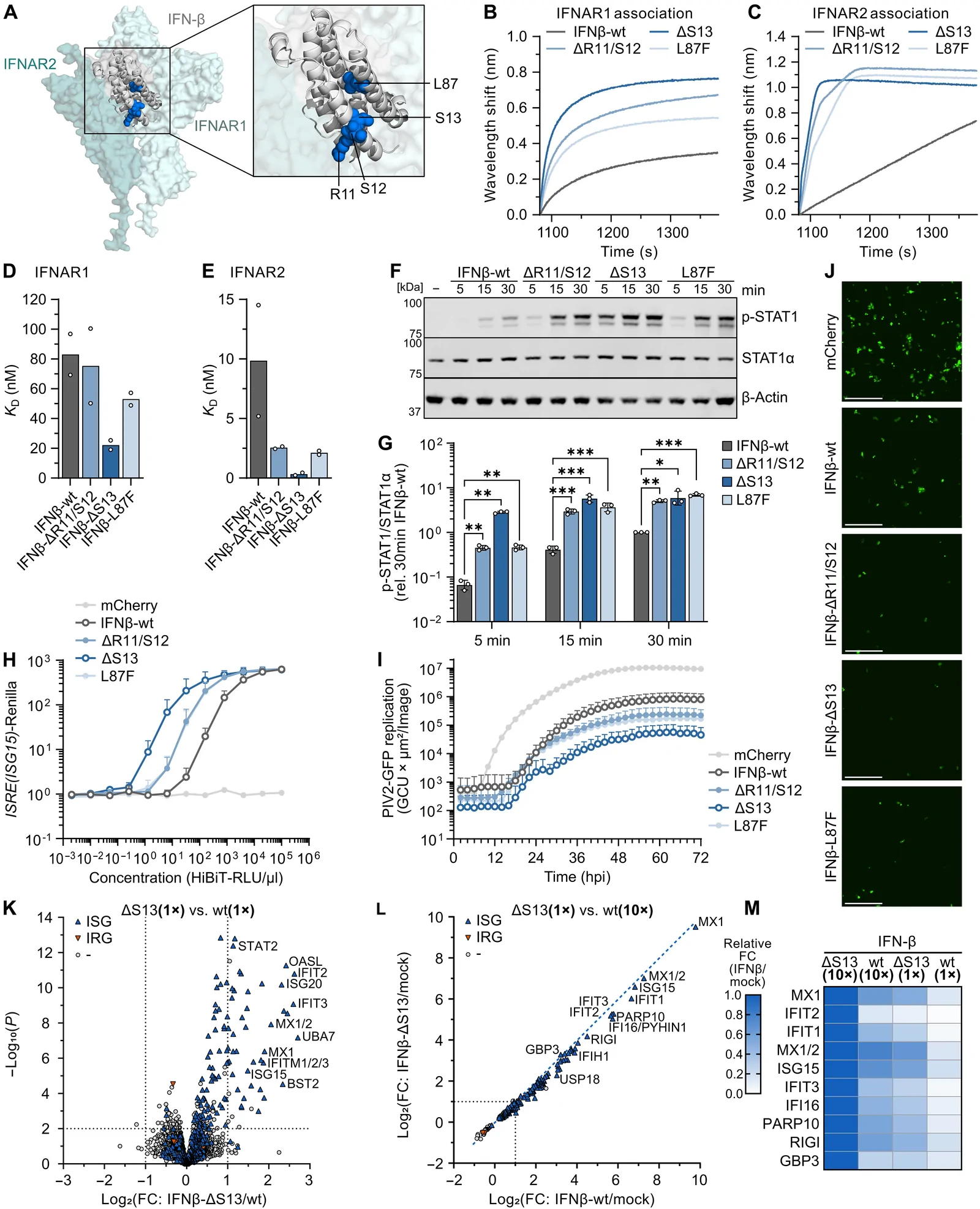
Highly potent IFN-β variants exhibit enhanced IFNAR1/2 receptor engagement and greater antiviral signaling. (**A**) Predicted structure of IFN-β with IFNAR1/2. More active variants are blue. (**B** and **C**) Binding of each HiBiT-tagged IFN-β, or mCherry, to IFNAR1/2 with BLI. Average wavelength shifts across *n* = 3 experiments shown. Replicates in fig. S4 (D to I). (**D** and **E**) Binding of each purified His-tagged IFN-β to IFNAR1/2 with BLI. *K*_D_s were determined across *n* = 2 experiments. Replicates in fig. S5 (A to P). (**F**) A549s were stimulated with each HiBiT–IFN-β as shown, and proteins were assessed by Western blot. (**G**) Quantification of STAT1 phosphorylation in (F) from *n* = 3 experiments ± SDs. Significance determined as in Fig. 4G. (**H**) AIR cells were stimulated with each HiBiT–IFN-β as in Fig. 4H. Means + SDs from *n* = 3 experiments. (**I**) A549s were stimulated with each HiBiT-tagged IFN-β and then infected with PIV2-GFP as in Fig. 4I. Data also in Fig. 4I (same experiments). (**J**) Representative images from (I) at 48 hours postinfection. Scale bars, 400 μm. (**K** to **M**) AIR cells were stimulated with 1× or 10× HiBiT quantities (4 × 10^3^ or 4 × 10^4^ HiBiT-RLU/μl) of each HiBiT-tagged IFN-β, or mock, for 24 hours before lysis and proteome assessment. Experiments conducted in *n* = 3 wells per condition and only proteins detected in all replicates are shown. (K) Differentially expressed proteins in IFNβ-ΔS13 over IFNβ-wt stimulated cells plotted against −log_10_(*P*). Significance was determined by a *t* test. (L) Significant (*P* < 0.01) differentially expressed proteins in IFNβ-wt(10×) stimulated cells over mock plotted against IFNβ-ΔS13(1×) over mock. ISGs and IFN-repressed genes (IRGs) with > two-fold change (*46*) are highlighted. (M) Heatmap shows relative fold change (FC) in abundance over mock for the top 10 up-regulated proteins after IFNβ-ΔS13(10×) stimulation. See data S2. Data also shown in fig. S6 (C and D).

We next focused on the three IFN-β variants that were identified in our initial phenotypic screen to be more active than IFNβ-wt. One variant consists of the single L87F substitution, while the other two variants, ΔR11/S12 and ΔS13, are notable for their deletions in a region of the mature IFN-β N-terminus (Fig. 5A). Human IFN-β consists of five α helices (commonly named A to E) (*43*). L87 is an inward-facing residue located in the C helix, and its substitution could affect IFNAR1 binding due to proximity of this helix to IFNAR1 (*6*, *44*, *45*). Both deletion variants are predicted to partially truncate the A helix (most N-terminal α helix) of IFN-β (fig. S4, A to C), which is in a region where both receptor chains, IFNAR1 and IFNAR2, are predicted to interact (Fig. 5A and fig. S4, A to C) (*6*, *44*, *45*). In BLI, all three IFN-β variants showed increased binding to both IFNAR1 and IFNAR2 compared to IFNβ-wt (Fig. 5, B and C, and fig. S4, D to M). Furthermore, using purified His-tagged IFN-β [wild type (wt) and variants] generated in Expi293F cells, as well as Fc-tagged IFNAR1 and IFNAR2, comprehensive BLI assays were used to determine that all three IFN-β variants exhibit increased affinities toward the receptors, with IFNβ-ΔS13 demonstrating the strongest binding and lowest dissociation constant (*K*_D_) (Fig. 5, D and E, and fig. S5). Consistent with increased IFNAR1/2 interactions, the IFN-β variants triggered STAT1 phosphorylation to a greater magnitude than IFNβ-wt when A549 cells were stimulated for 5 to 30 min with 2 × 10^4^ HiBiT-RLU/μl of each HiBiT-tagged IFN-β (Fig. 5, F and G). Notably, the IFNβ-ΔS13 variant induced the fastest and strongest STAT1 phosphorylation response, which was consistent with it also triggering the greatest *ISRE*-Renilla activation (Fig. 5H). Combining variants could not increase the potency of IFNβ-ΔS13 further (fig. S6A). In line with the results, 6-hour prestimulation of A549 cells with 1 × 10^5^ HiBiT-RLU/μl of IFNβ-ΔR11/S12, -ΔS13, or -L87F inhibited PIV2-GFP replication significantly more potently than IFNβ-wt, with IFNβ-ΔS13 being the most antivirally active (Fig. 5, I and J, and fig. S6B). We next applied mass spectrometry to assess directly the spectrum and magnitude of different ISG proteins that are induced by the highly potent IFNβ-ΔS13 as compared to IFNβ-wt. To this end, we stimulated AIR cells for 24 hours with 1× (4 × 10^3^ HiBiT-RLU/μl) or 10× (4 × 10^4^ HiBiT-RLU/μl) HiBiT quantities of IFNβ-ΔS13, -wt, or an untransfected cell culture supernatant control (mock). Consistent with our other assays, the same HiBiT quantity of IFNβ-ΔS13 triggered substantially higher expression of known ISG proteins (*46*) than IFNβ-wt (e.g., STAT2, IFIT2, ISG15, and MX1; Fig. 5K, fig. S6C, and data S2). In addition, we noted that stimulation with a 10× higher concentration of IFNβ-wt than IFNβ-ΔS13 led to ISG proteins being induced to similar magnitudes between the two conditions (Fig. 5, L and M, and data S2) and that there were no significant differentially stimulated proteins between the two IFN-βs (fig. S6D). These data reveal that naturally occurring IFN-β variants can exhibit greater than expected IFNAR1/IFNAR2 receptor engagement and can increase the kinetics and magnitude of canonical antiviral IFN-I signaling.

## DISCUSSION

IFN-β is an important part of innate immunity (*1*–*3*). Its integrity is crucial for orchestrating effective protection against viral pathogens while maintaining immune homeostasis. Here, we functionally assessed 70 naturally occurring IFN-β protein variants in the global human population and found that ∼25% of those tested are considerably less active than the common IFN-β. Furthermore, we identified three naturally occurring variants that exhibit substantially higher activity than the common IFN-β variant. All protein-altering *IFNB1* variants recorded in the gnomAD database are rare (<1% prevalence worldwide), and the allele frequencies of our studied variants range between 3 × 10^−6^ and 3 × 10^−3^. Eleven of the variants we tested have been observed to occur homozygously in at least one individual, including IFNβ-L133R that exhibits substantially diminished secretory and antiviral activity compared to the common IFN-β variant, as well as IFNβ-ΔR11/S12 that has enhanced activity. This homozygosity may mean that the biochemical phenotypes we describe could have clinical consequences, although none of our tested *IFNB1* variants have been previously associated with a pathogenic phenotype according to ClinVar (*47*). The rarity of these *IFNB1* variants might be one factor contributing to the lack of recorded pathogenicity at the population level to date. Nevertheless, one could speculate that functional deficiency in IFN-β caused by rare homozygous loss of function in some of the variants identified here, including L133R or others, may lead to disease outcomes such as increased susceptibility to certain infections in individuals, as has been reported for those with neutralizing autoantibodies against IFN-β or other IFN-Is (*22*, *48*, *49*). Furthermore, with the possibility of monoallelic IFN-β expression, which can predominate under certain conditions (*50*, *51*), as well as potential dominant-negative effects that we did not explore, pathogenic phenotypes may emerge even in those heterozygous for specific variants. Our functional work reported here might therefore serve as a useful resource for understanding whether any *IFNB1* genetic variants identified in individuals suffering from severe infections or other diseases are likely to underlie pathogenicity. Similarly, the biological consequences of IFN-β variants with apparently enhanced activities, such as ΔR11/S12, ΔS13, or L87F, could plausibly have pathogenic roles relating to autoinflammatory disorders (*23*) or potentially enhance resistance to infection. Our results complement recent similar systematic large-scale functional screens performed for human *IFNAR1* variants, which have created a reference to understand clinical profiles (*52*).

While our own mechanistic studies identified that some loss-of-function IFN-β variants were well secreted but nevertheless impaired due to their compromised interactions with the IFNAR1/2 receptors (e.g., L32P or Q72R), it was notable that many natural loss-of-function variants were either moderately or severely impaired in their secretory capacities (16 of 18: G26R, D34N, ΔE53, A55T, T58S, I59N, I59T, M62T, T77I, L88P, I95R, G114*, L133R, A142P, A142T, and I150Sfs*2). These phenotypes may not have been readily identified if the IFN-β variants had been expressed intracellularly in commonly used bacterial systems followed by denaturing purification and refolding (*53*), suggesting one experimental advantage to using human cells and the supernatant collection approach. Nevertheless, a limitation of our system, based on HiBiT quantification, is that all the IFN-βs retain a tag that could potentially interfere with optimal function. IFN-β is normally secreted via the conventional pathway through the ER and Golgi (*5*). During this process, correct protein folding is tightly monitored by the ER quality control machinery (*54*). For the poorly secreted IFN-β variants, we observed their notable ER retention, which is suggestive of their slower release from the ER and thus may indicate potential defects in correct protein folding, such as exposure of misfolded hydrophobic regions, or an impairment in glycosylation or disulfide bond formation (*55*). Beyond their own impaired secretion due to potential misfolding, it is also possible that ER accumulation of IFN-β variants could have other pathogenic consequences. For example, IFN-λ4, a type III IFN that is only expressed as a functional full-length protein in ∼30% of the European population (*56*), has been shown to accumulate in the ER where it impairs the processing and major histocompatibility complex loading of viral antigens and up-regulates some viral entry receptors (*40*, *41*). Furthermore, the intracellular accumulation of IFN-λ4 has been associated with ER stress and the unfolded protein response (UPR) (*40*, *41*), which can further diminish the secretion of other proteins and, if too severe, result in cell death (*57*). Thus, it is plausible that poorly secreted IFN-β variants may also activate the UPR and have broader dominant-negative consequences on other proteins that traffic through the ER.

It was intriguing to identify three IFN-β variants (ΔR11/S12, ΔS13, and L87F) that had greater activity compared to the common IFN-β variant. While potential positive or negative disease associations of these three variants are presently unclear, it might nevertheless be that understanding these naturally occurring highly potent variants could be informative in advancing the next-generation of IFN-β-based therapies against infections or MS. In addition, uncovering the principles underlying their heightened canonical action may have parallel applications for related IFN-Is, such as IFN-α2, that have also been used therapeutically. IFN-β displays the strongest antiviral activity among IFN-Is against different SARS-CoV-2 strains in vitro (*58*), and treatment with IFN-β increases survival rates after MERS-CoV infection (*10*). Together with multiple trials showing an effect of IFN-β therapy against SARS-CoV-2 (*11*–*15*), it is clear that even the widely used common IFN-β variant has potential as an effective antiviral therapeutic. New IFN-β–based therapeutics designed on the principles of the variants described here, such as ΔS13 which exhibits canonical responses, could be an even more beneficial therapeutic when strong and fast acting IFN-I signaling is required, as might be the case for rapid treatment of severe viral infections when the early timing of IFN-I–based treatments is critical (*8*). In addition, such activity-enhanced IFN-β therapies could be used to limit the development of resistance, which can occur due to emergence of neutralizing antidrug antibodies (*18*). In this context, potent activity–enhanced IFN-β would allow lower amounts of the protein to be given, limiting the chances of antidrug antibody responses.

In summary, our functional assessment of 70 naturally occurring human IFN-β protein variants now provides a resource for potentially understanding future disease susceptibility or resistance in individuals identified with *IFNB1* genetic variants. Furthermore, our identification of natural IFN-β variants with enhanced activities, as well as the mechanistic principles underlying their activities, may aid in optimizing future IFN-β (or other IFN-I)–based therapies in infectious or autoimmune diseases.

## MATERIALS AND METHODS

### Human genetic variant information

Genetic variant information was acquired from the Genome Aggregation Database (gnomAD v4.1, https://gnomad.broadinstitute.org/, October 2024). Briefly, variants were filtered to pass quality control and to be protein altering (including missense, nonsense, in-frame deletion, and frameshift variants and excluding synonymous, untranslated region, start lost, and stop retained variants). PHRED-scaled CADD (v1.7, https://cadd.bihealth.org/, March 2026) scores were obtained by manually uploading the *IFNB1* genetic variants. See data S1.

### Cells

HEK293T cells, human lung epithelial A549 cells (both originally from American Type Culture Collection), and A549 cells expressing Renilla luciferase under control of the human *ISG15* promoter (*38*) (AIR cells) were cultured at 37°C and 5% CO_2_ in Dulbecco’s modified Eagle’s medium (DMEM; Thermo Fisher Scientific), supplemented with 0 to 10% fetal bovine serum (FBS) and penicillin-streptomycin (100 U/ml; Gibco Life Technologies). Expi293F cells were cultured at 37°C, 8% CO_2_, and 160 rpm in Expi293 Expression Medium (Thermo Fisher Scientific) supplemented with penicillin-streptomycin (100 U/ml; Gibco Life Technologies).

### Plasmids

cDNA of the common full-length human *IFNB1* allele including its signal peptide (ENST00000380232.4, termed wt) with additional nucleotides encoding a C-terminal HiBiT tag was obtained via GeneArt Custom Gene Synthesis (Thermo Fisher Scientific) cloned into pCDNA3.1. cDNA encoding mCherry was amplified from p3xFlag-mCherry-CMV-7.1 (*59*), and nucleotides encoding a C-terminal HiBiT tag were appended using an appropriate reverse primer. mCherry-HiBiT was then cloned into pCDNA3.1 via restriction digest cloning using BamHI and XhoI. *IFNB1* variants (including the C-terminally truncated variants with distinct tag-encoding primers) were generated from the common *IFNB1* plasmid with QuikChange XL Site-Directed Mutagenesis (Agilent) according to the manufacturer’s recommendation. Two *IFNB1* variants, c.102C>G and c.102C>A, both encode for the same S13R substitution, and so only c.102C>G was generated. The plasmid encoding HiBiT-tagged simIFNα2 was previously described (*36*). His-tagged *IFNB1* variants were generated from the HiBiT-tagged ones by exchanging sequences encoding the C-terminal HiBiT-tag with the His_6_-tag sequence using QuikChange XL Site-Directed Mutagenesis (Agilent) according to the manufacturer’s recommendation. All protein-coding sequences were authenticated by Sanger sequencing (Microsynth).

### HEK293T transfections

HEK293T cells were transfected at 50 to 80% confluency using FuGENE HD transfection reagent (Promega) at a ratio of 3:1 (FuGENE:DNA) according to the manufacturer’s recommendation. Cells were transfected in DMEM with 10% FBS (for immunofluorescence microscopy assays), DMEM with 2% FBS (when generating supernatants for luciferase and virus reporter assays), or DMEM without FBS (when generating supernatants for BLI). Where appropriate, BFA (Sigma-Aldrich) was used at a concentration of 1 μg/ml for 6 hours, and MG132 (Selleck) was used at a concentration of 20 μM for 6 hours.

### HiBiT detection

HiBiT-tagged protein quantities were measured by Nano-Glo HiBiT Lytic Detection Assay (*37*) (Promega) according to the manufacturer’s recommendation. Briefly, undiluted or 1:100-diluted samples were incubated with the HiBiT reagents for 10 min at room temperature, and HiBiT signal was detected with an EnVision 2104 plate reader (PerkinElmer).

### Luciferase reporter assays

AIR cells were seeded at a density of 3 × 10^4^ cells per well in white 96-well plates. After 24 hours, cells were stimulated with a 1:5 dilution series of HiBiT-normalized quantities of the respective transfected HEK293T supernatant or cell lysate containing HiBiT-tagged IFN-β (wt or variants), mCherry, or simIFNα2 in DMEM containing 2% FBS and 1:10,000 EnduRen Live Cell Substrate (Promega). Six or 15 hours post stimulation, Renilla signal was quantified with an EnVision 2104 plate reader (PerkinElmer).

### Immunofluorescence microscopy

HEK293T cells were seeded on poly-l-lysine–coated coverslips at a density of ∼50% in 24-well plates and transfected as described above. Twenty-four hours posttransfection, cells were washed three times with phosphate-buffered saline (PBS) and fixed with 4% (w/v) paraformaldehyde in PBS for 10 min, before being washed again three times with PBS, permeabilized with 0.2% (v/v) Triton X-100 in PBS for 10 min, and then washed three times with PBS before blocking with 5% normal goat serum in PBS for 1 hour. Samples were then incubated as indicated with mouse anti-HiBiT antibody (Promega, N7200), rabbit anti-calnexin (Abcam, ab22595), or rabbit anti-giantin (Abcam, ab80864) in 5% normal goat serum in PBS for 2 hours. Coverslips were then stained with 4′,6-diamidino-2-phenylindole, Alexa Fluor 488 anti-rabbit immunoglobulin G (IgG), and Alexa Fluor 555 anti-mouse IgG (Thermo Fisher Scientific, A21206 and A31570) in 5% normal goat serum in PBS for 2 hours. Samples were mounted using ProLong Gold Antifade Mountant (Thermo Fisher Scientific) and imaged with a Leica SP8 upright confocal microscope (Leica Microsystems) using the LAS X software. Colocalization was assessed with a pixel-based approach to quantifying the spatial relationship between HiBiT and calnexin or between HiBiT and giantin. Following global intensity normalization of the images, the Pearson correlation coefficient was determined as a measure of signal association. The analysis was conducted using a custom Python script to ensure reproducible quantification across all experimental conditions.

### His-tagged IFN-β (wt and variant) protein expression and purification

Expi293F cells were transfected with 1 μg of His_6_-tagged IFN-β (wt or variant) expressing plasmid per 1 ml of culture using ExpiFectamine 293 (Thermo Fisher Scientific) according to the manufacturer’s instructions or using PEI MAX (Polysciences) at a ratio of 3:1 (PEI MAX:DNA). The protein purification protocol was adjusted from a prior study (*60*). Briefly, supernatants were harvested 72 hours posttransfection by centrifugation at 4000*g* at 4°C for 30 min and were then filtered through a 0.22-μm PES membrane. Flow-throughs were then mixed 1:1 with Ni-NTA loading buffer [50 mM PBS and 300 mM NaCl (pH 8.5)] and incubated with 1 ml of PBS-washed Ni-NTA Agarose (QIAGEN) per 40 ml of flow-through overnight at 4°C. The Ni-NTA agarose was loaded onto gravity flow columns, washed four times with Ni-NTA wash buffer [50 mM PBS, 300 mM NaCl, and 20 mM imidazole (pH 8.0)], and eluted by incubating four times for 5 min with Ni-NTA elution buffer [50 mM PBS, 300 mM NaCl, and 250 mM imidazole (pH 8.0)]. The eluate buffer was exchanged by repeatedly centrifuging in PBS-equilibrated Pierce Protein Concentrators (PES, 3K MWCO, Thermo Fisher Scientific) at 4000*g* at 4°C until <500 μl remained, and then 1× PBS (pH 7.5) was added as a dilution step. Protein concentration was determined with the Pierce BCA Protein Assay Kit (Thermo Fisher Scientific) according to the manufacturer’s recommendations. His-tagged IFN-βs (wt or variant) were diluted to the same concentrations in 1× PBS (pH 7.5). Recombinant protein molecular weight and purity were regularly assessed in samples taken throughout the purification procedure by SDS–polyacrylamide gel electrophoresis and Coomassie Blue staining.

### Biolayer interferometry

The His-tagged extracellular domains of IFNAR1 (IF1-H5225; ACROBiosystems) or IFNAR2 (IF2-H5224; ACROBiosystems) were diluted in 1× PBS (pH 7.5) containing 0.1% bovine serum albumin (BSA) and 0.02% Tween 20 as described previously (*36*), and the indicated HiBiT-tagged proteins were diluted to the same concentrations (2 × 10^7^ or 5 × 10^7^ RLU/μl as required) in DMEM without FBS. All reagents were pipetted into a Greiner black microplate, and prehydrated Ni-NTA biosensors (18-5101; Sartorius) were used to perform standard kinetic analyses with an Octet R2 (Sartorius). Times were as follows: 120-s baseline 1 in 1× PBS (pH 7.5) containing 0.1% BSA and 0.02% Tween 20, 600-s loading of His-tagged IFNAR1/2, 360-s baseline 2 in DMEM, 300-s association, and 300-s dissociation. For the experiments shown in fig. S3 (J to M), association and dissociation times were extended to 360-s each. Association and dissociation were normalized to mCherry containing samples (negative control). Area under the curve (AUC) of the association phase was calculated in GraphPad Prism 10 and normalized to IFNβ-wt.

For BLI experiments using purified His-tagged IFN-β (wt or variants), Fc-tagged extracellular domains of IFNAR1 (IF1-H5253; ACROBiosystems) or IFNAR2 (IF2-H5255; ACROBiosystems) were diluted in 1× PBS (pH 7.5) containing 0.1% BSA and 0.02% Tween 20. All reagents were pipetted into a Greiner black microplate, and prehydrated anti-Human Fc Capture biosensors (18-5060; Sartorius) were used to perform standard kinetic analyses with an Octet R2 (Sartorius). Concentrations of 500, 100, 20, and 4 nM His-tagged IFN-β proteins [in 1× PBS (pH 7.5) containing 0.1% BSA and 0.02% Tween 20] were assessed for binding to IFNAR1, while 10-fold lower concentrations were applied to assess IFNAR2 binding. Times were as follows: 120-s baseline 1 in 1× PBS (pH 7.5) containing 0.1% BSA and 0.02% Tween 20, 480-s loading of 80 nM Fc-tagged IFNAR1/2, 300-s baseline 2 in 1× PBS (pH 7.5) containing 0.1% BSA and 0.02% Tween 20, 360-s association, and 360-s dissociation. *K*_D_s were determined with the Octet Analysis Studio 13.0 (Sartorius) using a 1:1 model and a grouped fitting for the different His-tagged IFN-β protein concentrations.

### PIV2-GFP reporter assays

A total of 1.6 × 10^4^ A549 cells were seeded in wells of a 96-well plate and infected 24 hours later with PIV2-GFP (MOI = 0.1 PFU per cell; originally from ViraTree) in DMEM containing 2% FBS. Cells were monitored with the IncuCyte live-cell imaging system (Sartorius) for 72 hours, and total integrated intensities (green calibrated unit × μm^2^/image) were determined.

### Western blotting

Cells were lysed in 1× Laemmli [4× Laemmli (Bio-Rad) with β-mercaptoethanol, diluted to 1× in water]. Samples were boiled at 95°C for 5 min and loaded onto Bolt 4 to 12% Bis-Tris Plus Gels (Thermo Fisher Scientific). Proteins were transferred onto 0.45-μm nitrocellulose membranes according to the manufacturer’s recommendation. Blocking and antibody incubations were done in 5% milk in tris-buffered saline (TBS) containing 0.1% Tween 20. Membranes were blocked for 1 hour at room temperature and incubated overnight at 4°C with the primary antibodies, mouse anti–β-actin (Santa Cruz, sc-47778), rabbit anti-actin (Sigma-Aldrich, A2103), mouse anti-STAT1α (Santa Cruz, sc-417), rabbit anti–phospho-STAT1 (Cell Signaling Technology, 9167), mouse anti-HiBiT (Promega, N7200), or mouse anti-ubiquitin (P4D1; Cell Signaling Technology, 3936). After washing in TBS containing 0.1% Tween 20, membranes were incubated for 1 hour at room temperature with the secondary antibodies, IRDye 800CW goat anti-mouse IgG (LI-COR Biosciences, 926-32210), or IRDye 680RD goat anti-rabbit IgG (LI-COR Biosciences, 926-68071). After washing in TBS containing 0.1% Tween 20, an Odyssey Fc Imager and the Image Studio Lite Quantification software (LI-COR Biosciences) were used for signal detection and quantification.

### Mass spectrometry lysate preparation and processing

AIR cells were cultured to 90% confluency in a six-well plate before stimulation with 1× (4 × 10^3^ HiBiT-RLU/μl) or 10× (4 × 10^4^ HiBiT-RLU/μl) HiBiT quantities of HEK293T supernatant–derived HiBiT-tagged IFNβ-wt or IFNβ-ΔS13. The IFNs were first diluted in the HEK293T cell supernatant from untransfected cells to control for any possible effect of the supernatant and then further diluted 100-fold to the working concentration by adding 20 μl to 1980 μl of DMEM containing 2% FBS. Mock-treated cells received 20 μl of the mCherry-expressing HEK293T supernatant. Five conditions were tested in triplicates: mock, IFNβ-wt (1×), IFNβ-wt (10×), IFNβ-ΔS13 (1×), and IFNβ-ΔS13 (10×).

Twenty-four hours poststimulation, the supernatant was removed, and the cells were washed twice with PBS before adding 500 μl of lysis buffer. The lysis buffer contained 1% SDS, 50 mM tris, 150 mM NaCl, 1% Triton X-100, 0.5% sodium deoxycholate, and 1× protease inhibitors (pH 8). Cell lysates were sonicated before the soluble fraction was isolated by centrifugation at 4°C and 18,000*g* for 30 min and then frozen at −80°C. Protein concentration of the soluble fraction was determined using BCA assay (Thermo Fisher Scientific, 23225). Samples containing 50 μg of protein were diluted to equal volumes with lysis buffer, then reduced with 2 mM tris(2-carboxyethyl)phosphine, and alkylated with 15 mM chloroacetamide for 30 min at 60°C while shaking at 700 rpm in the dark. Samples were further prepared using the single-pot solid-phase enhanced sample preparation (SP3) (*61*) workflow and carboxylate-modified magnetic beads (GE Life Sciences, GE65152105050250 and GE45152105050250). Following the manufacturer’s instructions, ethanol was added to each sample to a final concentration of 60% (v/v), after which proteins were captured using a 1:1 mixture of the two types of carboxylate-modified magnetic beads. Samples were then incubated for 30 min at room temperature with shaking at 800 rpm to allow efficient protein binding. Beads were washed twice with 80% ethanol and then resuspended in 100 μl of 50 mM triethylammonium bicarbonate (pH 8) containing 0.3 μg of trypsin (Promega, V5113). Proteins were digested overnight at 37°C at 800 rpm before the peptide-containing supernatant was collected, and the beads were washed once more with 100 μl of ddH_2_O. Both the overnight digest and ddH_2_O wash samples were merged and acidified with TFA to an end concentration of 0.5%. C18 StageTips were used for peptide cleanup. Tips were wetted with 100% ACN, equilibrated with 60% ACN containing 0.1% TFA, and conditioned twice with 3% ACN containing 0.1% TFA. All steps used 150 μl and were followed by centrifugation at 2000*g*. Digested peptides were loaded onto the StageTips and centrifuged at 2000*g*. Bound peptides were washed three times with 3% ACN containing 0.1% TFA and eluted with 60% ACN containing 0.1% TFA. Eluates were dried in a SpeedVac before reconstitution in 20 μl of 3% ACN with 0.1% formic acid and stored at −20°C.

### Mass spectrometry data acquisition and analysis

Mass spectrometry analysis was performed on a timsTOF Pro HT (Bruker) coupled to an Evosep One (EvoSep Biosystems). Samples were separated with the Evosep method “30 SPD” and the analytical column (PepSep C18, 15 cm by 150 μm, 1.5 μm) at 40°C. For the dual timsTOF, MS spectra were scanned from mass/charge ratio (*m/z*) 100 to *m/z* 1700 and inverse mobilities [1/*K*_0_] from 0.60 to 1.60 Vs/cm^2^ with an ion accumulation and ramp time of 100 ms, respectively. The data were acquired in diaPASEF mode (data-independent acquisition Parallel Accumulation Serial Fragmentation); 1 MS scan was followed by 16 PASEF cycles between *m/z* 400 and *m/z* 1200 with overlapping isolation windows of *m/z* 26, covering with 2 × 0.30 [1/*K*_0_] windows the ion mobility range of 0.60 to 1.42 Vs/cm^2^. Singly charged ions were excluded using the polygon filter mask.

The mass spectrometry proteomic data were handled using the local laboratory information management system (*62*). All data have been deposited to the ProteomeXchange Consortium via the PRIDE (http://ebi.ac.uk/pride) partner repository with the dataset identifier PXD076103. Data were analyzed using DIA-NN version 1.9.2, and searches were performed against a human reference proteome (March 2023). Precursor charge states were restricted to 2/3. Precursor and fragment mass ranges were set to 400 to 1500 *m/z* and 200 to 1800 *m/z*, respectively. A maximum of one missed cleavage was allowed. Mass tolerances were set to 15 parts per million (ppm) for MS1 and 20 ppm for MS2.

Differential expression analysis was performed using the R packages prolfqua (*63*) and prolfquapp (*64*) which handle statistical modeling of proteomic data and provide estimates of confidence intervals and false discovery rates for quantified proteins. Within this workflow, protein abundances were inferred from peptide intensities in the DIA-NN report.tsv using Tukey’s median polish, followed by robscale standardization to reduce outlier effects before linear modeling. Linear models were fitted for differential expression analysis, and *P* values were obtained from *t* tests on model coefficients. Identified proteins were matched against a previous dataset to identify ISGs and IFN-repressed genes (*46*). Significantly different proteins were defined as those exhibiting log_2_(fold change) > 1 and −log_10_(*P* value) > 2 that were detected in all three replicates after IFNβ-wt and IFNβ-ΔS13 stimulation. See data S2.

### Structure prediction

The structure of mature human IFN-β variants or mature human IFN-β in complex with extracellular IFNAR1 (K1-I409, numbering excluding the signal peptide) and extracellular IFNAR2 (I1-I217, numbering excluding the signal peptide) was predicted with ColabFold (*44*, *45*) v1.5.5 (December 2025). Structure visualizations were generated in PyMOL (*65*) v.2.4.1.

### Statistical analyses

Statistical analyses were performed in GraphPad Prism 10. Data were analyzed by a two-tailed unpaired *t* test with Welch’s correction (BFA assay), a Kruskal-Wallis test with Dunn’s multiple comparisons (Pearson correlation), a one-sample *t* test toward 0 on the log_10_-transformed values (normalized BLI association AUC, normalized PIV2-GFP signal), a one-sample *t* test toward 1 (BLI input Western blot quantification), two-way analysis of variance (ANOVA) with Dunnett’s multiple comparisons test on the log_10_-transformed values [*ISRE(ISG15)*-Renilla data, p-STAT1/STAT1 Western blot quantification].
