# Supplementary material for: Functional diversity of natural human IFNB1 variants in innate immunity
Source: Sci Adv. 2026 Sep 18;12(38):eaeh4755. doi: 10.1126/sciadv.aeh4755 (PMC13588187; doi:10.1126/sciadv.aeh4755)
Supplement: Supplementary file 1 [file sciadv.aeh4755_sm.pdf]

Science Advances
AAAS

Supplementary Materials for

Functional diversity of natural human *IFNB1* variants in innate immunity

Samira Schiefer *et al.*

Corresponding author: Benjamin G. Hale, hale.ben@virology.uzh.ch

Sci. Adv. **12**, eaeh4755 (2026)
DOI: 10.1126/sciadv.aeh4755

The PDF file includes:

Figs. S1 to S6
Legends for data S1 to S3

Other Supplementary Material for this manuscript includes the following:

Data S1 to S3

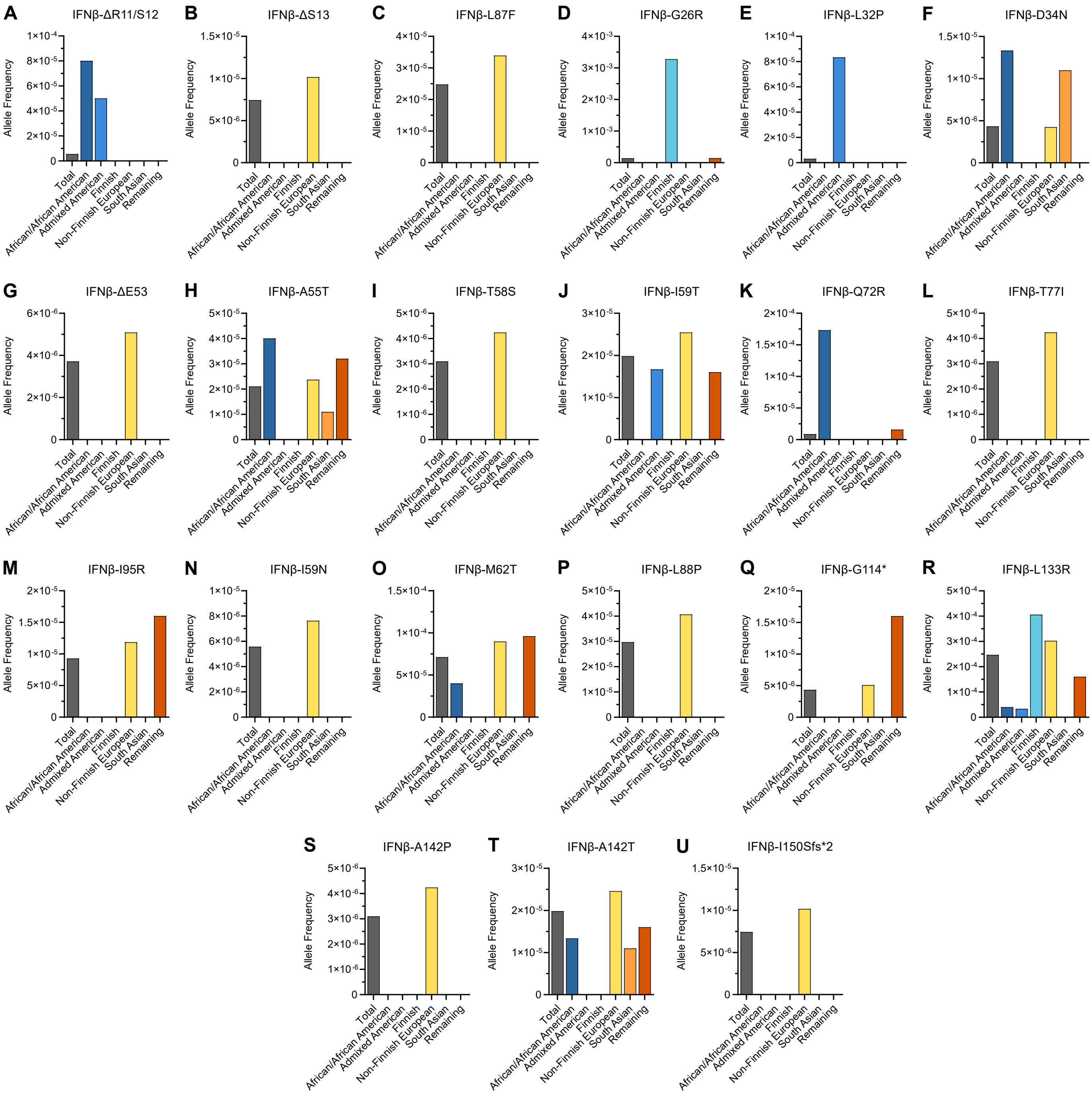

Fig. S1: Allele frequencies in different human populations for the more active, less active, or not/barely active protein-altering IFNβ variants.

A-C more active, **D-M** less active, and **M-U** not/barely active variants, and their allele frequencies in the indicated populations. More detailed information can be found in Data S1 and Fig. 2.

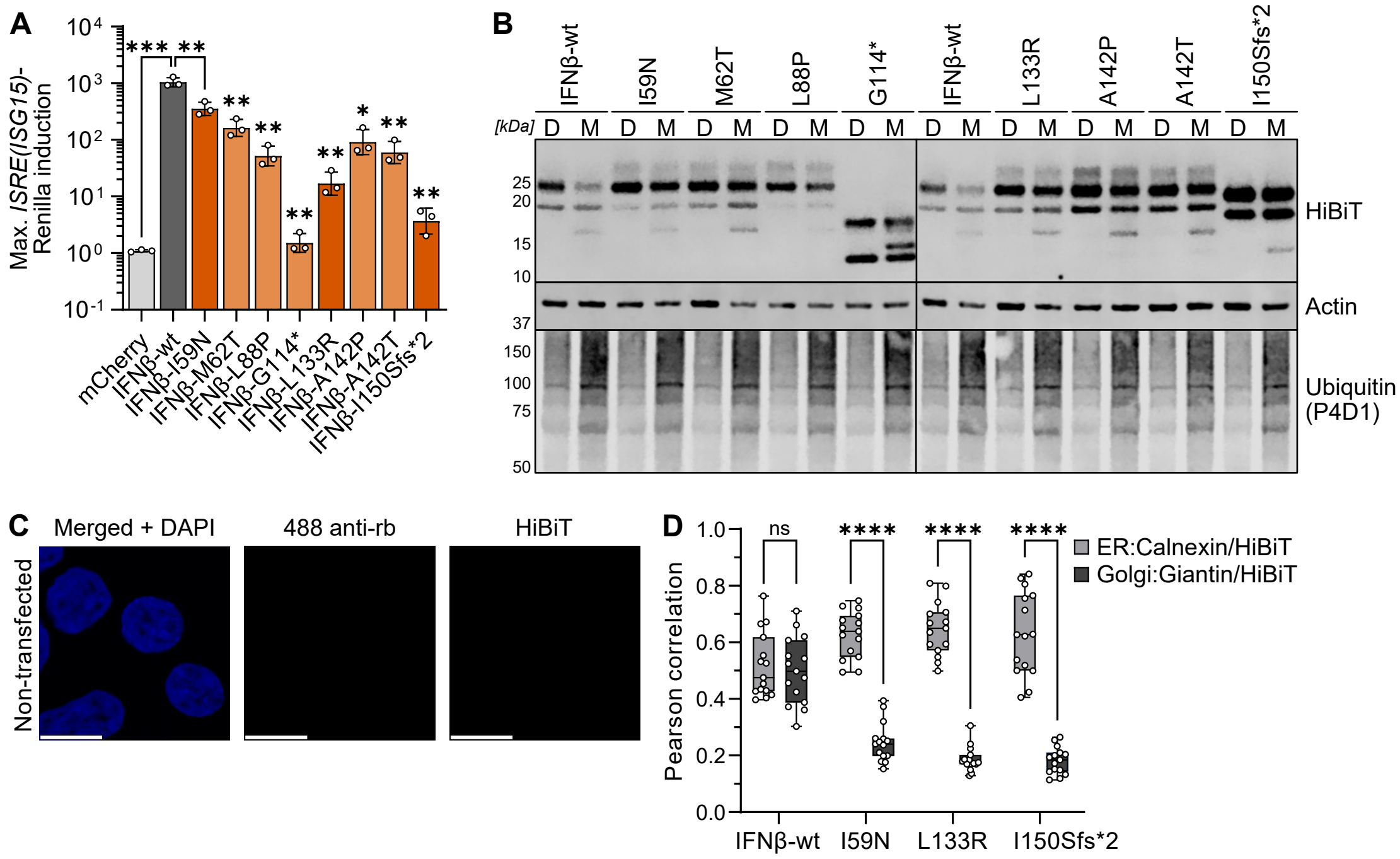

Fig. S2: Activity of poorly secreted variants and their ER retention.

A AIR cells were stimulated for 15 h with equal quantities of HEK293T cell lysate-derived HiBiT-tagged IFNβ (wt or variants), or mCherry, in a 1:5 dilution series. Maximal *ISRE(ISG15)*-Renilla induction over background is depicted. Mean values ± SDs from n=3 independent experiments are shown. Significance towards IFNβ-wt was determined by one-way ANOVA with Dunnett's multiple comparisons test on the $\log_{10}$-transformed values (* $P < 0.05$; ** $P < 0.01$; *** $P < 0.001$). Data are also shown in Fig. 3, B and C. **B** HEK293T cells were transfected with plasmids expressing HiBiT-tagged IFNβ (wt or variants) for 24 h before being treated with 20µM MG132 (or a DMSO control) for 6 h. Levels of the indicated proteins were determined by western blot. **C** Non-transfected HEK293T cells were assessed for background staining (Alexa Fluor 488 anti-rabbit and HiBiT / Alexa Fluor 555 anti-mouse) via immunofluorescence (IF) imaging. Representative IF images are shown. Scale bar, 10 µm. **D** HEK293T cells were transfected to express HiBiT-tagged IFNβ (wt or variants) or mCherry. The co-localization (Pearson correlation coefficient) of endoplasmic reticulum (ER: Calnexin) or Golgi (Giantin) with HiBiT signal was assessed by IF microscopy (see Fig. 3). Single dots represent single images with at least 10 cells. N=5 images were analyzed for n=3 independent replicates (all data shown in the plots). Significance was determined by two-way ANOVA with Šídák's multiple comparisons test (**** $P < 0.0001$; ns, non-significant). Data are also shown in Fig. 3, E to H.

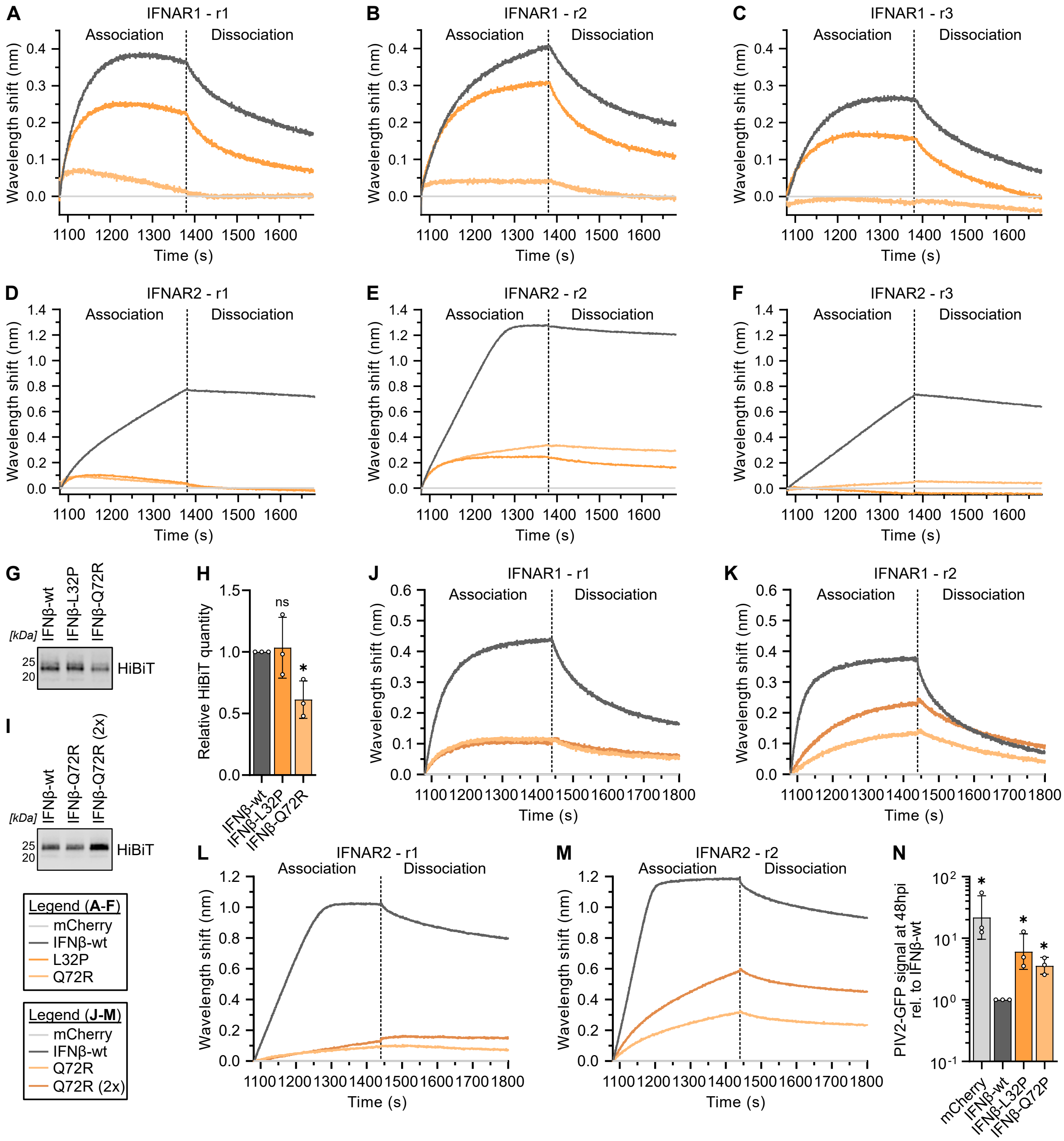

Fig. S3: Selected low potency IFNβ variants exhibit impaired IFNAR1/IFNAR2 receptor engagement and diminished antiviral activity.

A-F Complete single replicate BLI measurements (association and dissociation) of the data shown in Fig. 4B (A to C) and Fig. 4C (D to F). Equal quantities (5 x 10^7 HiBiT-RLU/µl) of HiBiT-tagged IFNβ (wt or variants), or mCherry, were assessed for binding to IFNAR1 and IFNAR2. **G** Levels of the HiBiT-tagged IFNβ variants used in the BLI (Fig. 4, B to E, fig. S3, A to F) as determined by western blot. Data shown are representative of n=3 independent batches and assays.

H Quantification of HiBiT-tagged IFNβ variant levels in G from the n=3 independent assays. Mean values ± SDs are shown. Significance was determined by one sample t test towards 1 (* $P < 0.05$; ns, non-significant). **I** Levels of the HiBiT-tagged IFNβ variants used in the BLI (fig. S3, J to M) as determined by western blot. Data shown are representative of n=2 independent batches and assays. **J-M** Single replicate BLI measurements (n=2 independent experiments, association and dissociation) of equal quantities (5×10^7 HiBiT-RLU/μl) of HiBiT-tagged IFNβ (wt or Q72R), or mCherry, or 2x as much of the Q72R variant (1×10^8 HiBiT-RLU/μl), as assessed for binding to IFNAR1 and IFNAR2. **N** A549 cells were stimulated with equal quantities (1×10^5 HiBiT-RLU/μl) of HEK293T supernatant-derived HiBiT-tagged IFNβ (wt or variants, normalized by Nano-Glo HiBiT Lytic Detection Assay), or mCherry, for 6 h prior to infection with PIV2-GFP (MOI = 0.1 PFU/cell). Cells were imaged with the IncuCyte system and mean values of the GFP signal (48 hpi) ± SDs are shown relative to IFNβ-wt results. Data from n=3 independent experiments are depicted. Significance was determined by one sample t test towards 0 on the $\log_{10}$-transformed values (* $P < 0.05$; ** $P < 0.01$). Data derive from experiments shown in Fig. 4I.

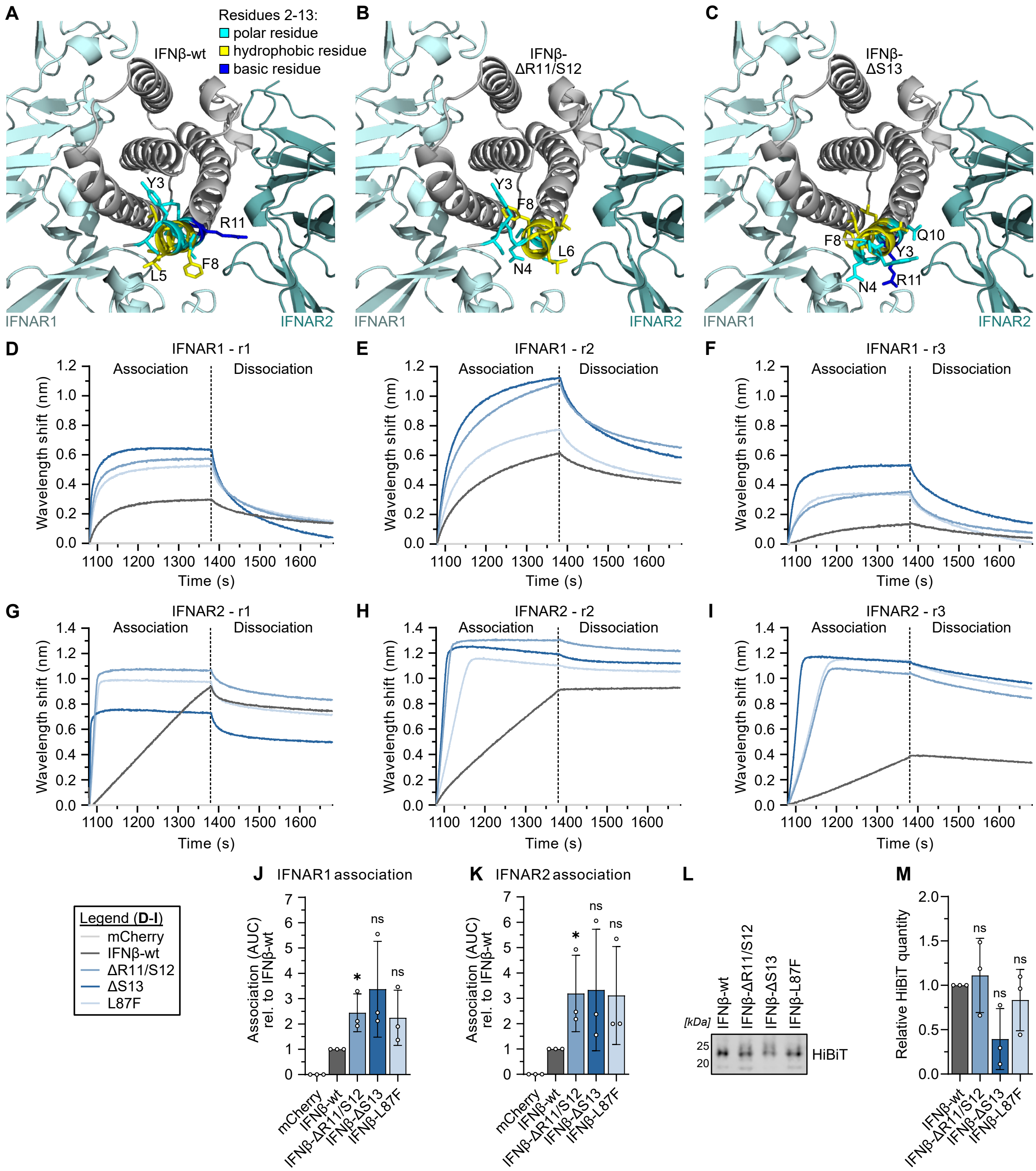

Fig. S4: Characterization of highly potent IFNβ variants and their enhanced IFNAR1/ IFNAR2 receptor engagement.

A-C Structure of IFNβ-wt (**A**) or the more active deletion variants (**B, C**) as predicted with ColabFold [9,10] v1.5.5 and in complex with IFNAR1/2. Amino acid residues N-terminal of the variant deletions are annotated and colored by property to highlight potential physical or structural

changes. **D-I** Complete single replicate BLI measurements of the data shown in Figs. 5, B and C. Equal quantities (2 x 10^7 HiBiT-RLU/µl) of HiBiT-tagged IFNβ (wt or variants), or mCherry, were assessed for binding to IFNAR1 and IFNAR2. **J-K** Equal quantities (2 x 10^7 HiBiT-RLU/µl) of HiBiT-tagged IFNβ (wt or variants), or mCherry, were assessed for binding to IFNAR1 and IFNAR2 with BLI. The AUC ± SDs during association across n=3 independent replicates are shown. Significance was determined by one sample t test towards 0 on the $\log_{10}$-transformed values (* $P < 0.05$; ns, non-significant). Data derive from the experiments shown in Figs. 5, B and C and Fig. S4, D to I. **L** Levels of the HiBiT-tagged IFNβ variants used in the BLI assay (Fig. 5, B and C, fig. S4, D to I) were determined by western blot. Data shown are representative of n=3 independent batches and assays. **M** Quantification of HiBiT levels in L from the n=3 independent assays. Mean values ± SDs are shown. Significance was determined by one sample t test towards 1 (ns, non-significant).

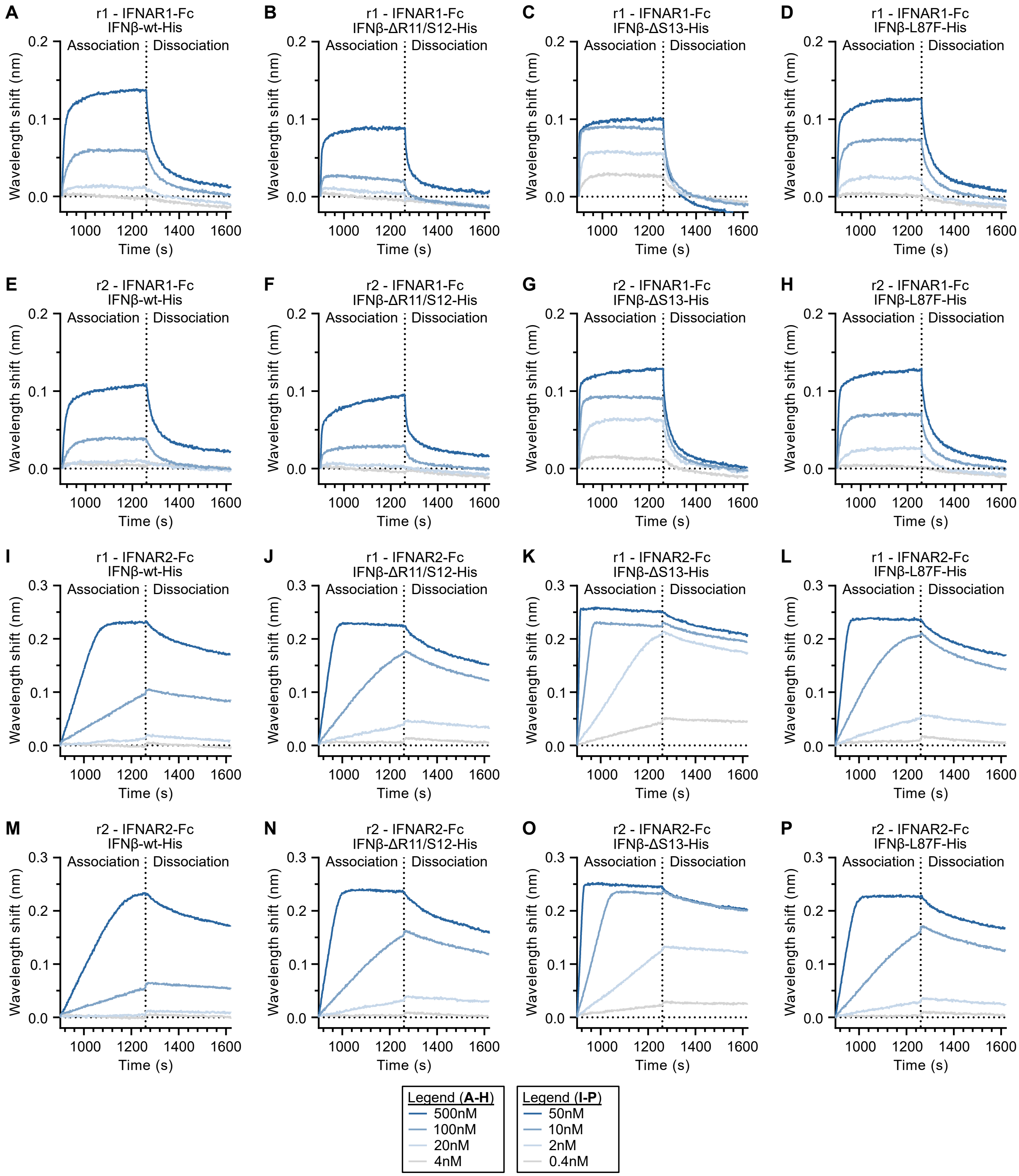

Fig. S5: Characterization of purified recombinant highly potent IFNβ variants.

A-P Single replicate BLI measurements (Savitzky-Golay-filtered association and dissociation) of the data summarized in Fig. 5, D and E. 500, 100, 20, and 4 nM of His-tagged IFNβ (wt or variants) were assessed for binding to IFNAR1 (**A-H**) and 50, 10, 2, and 0.4 nM of His-tagged IFNβ (wt or variants) were assessed for binding to IFNAR2 (**I-P**). r1 or r2 indicates the independent replicates.

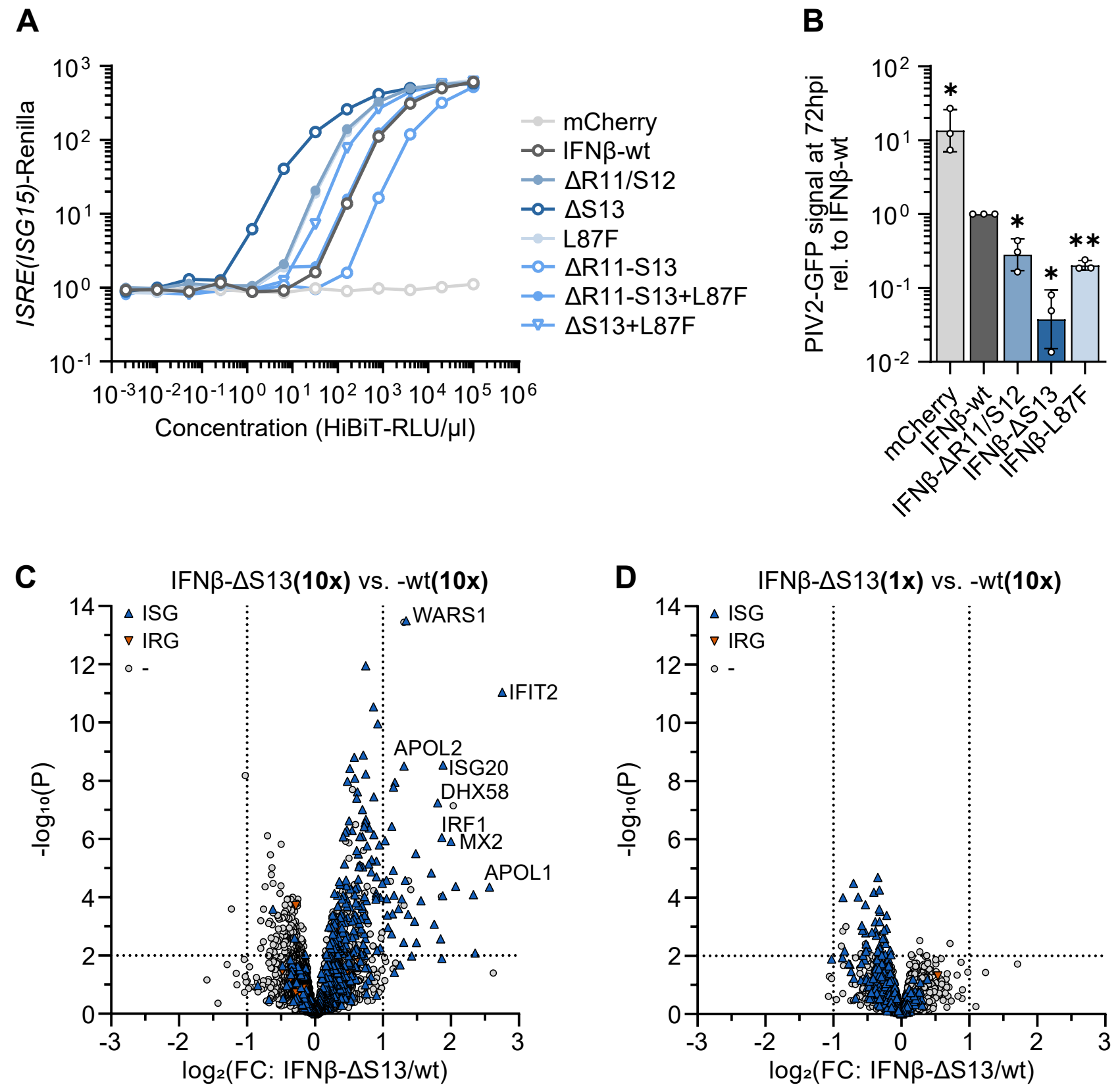

Fig. S6: Characterization of highly potent IFNβ variants and their enhanced antiviral signaling capacities.

A AIR cells were stimulated for 6 h with equal quantities of HEK293T supernatant-derived HiBiT-tagged IFNβ (wt or indicated variants), or mCherry, in a 1:5 dilution series. *ISRE(ISG15)*-Renilla induction over background is plotted versus HiBiT concentration. Mean values from n=2 independent experiments are shown. Note: mCherry, IFNβ-wt, IFNβ-ΔR11/S12, IFNβ-ΔS13, IFNβ-L87F data are also shown in Fig. 5H. **B** A549 cells were stimulated with equal quantities (1×10^5 HiBiT-RLU/µl) of HEK293T supernatant-derived HiBiT-tagged IFNβ (wt or variants), or mCherry, for 6 h prior to infection with PIV2-GFP (MOI = 0.1 PFU/cell). Cells were imaged with the IncuCyte system and mean values of the GFP signal (72 hpi) ± SD are shown relative to IFNβ-wt results. Data from n=3 independent experiments are depicted. Significance was determined by one sample t test towards 0 on the $\log_{10}$-transformed values (***** $P < 0.05$; ****** $P < 0.01$). Data derive from the experiments shown in Fig. 5I. **C-D** AIR cells were stimulated with 1x or 10x HiBiT quantities (4×10^3 or 4×10^4 HiBiT-RLU/µl) of HEK293T supernatant-derived HiBiT-tagged IFNβ (wt or ΔS13) for 24 h prior to cell lysis and total proteome assessment via mass spectrometry. All differentially expressed proteins in IFNβ-ΔS13(10x, **C**; or 1x, **D**) over IFNβ-wt(10x) stimulated cells are plotted against $-\log_{10}(P)$. Significance was determined by t test. Experiments were conducted in n=3 separate wells per condition and only proteins detected in all 3 replicates are shown. IFN-stimulated genes (ISGs) and IFN-repressed genes (IRGs) with a >2-fold change are highlighted. Data derive from the experiments shown in Fig. 5, K to M.

Data S1. (separate file)

IFNB1 genetic variants. Overview of all *IFNB1* genetic variants that affect the protein-coding sequence, the different *IFNB1* genetic variants that were assessed in the present study, and information on the populations in which more and less active *IFNB1* genetic variants were identified.

Data S2. (separate file)

Total proteome assessment of IFNβ-wt or -ΔS13 stimulated AIR cells via mass spectrometry. AIR cells were stimulated with 1x or 10x HiBiT quantities (4 x 10^3 or 4 x 10^4 HiBiT-RLU/µl) of HEK293T-supernatant derived IFNβ-wt, -ΔS13, or an untransfected supernatant control (mock) for 24 h prior to cell lysis and mass spectrometry. Experiments were conducted in n=3 separate wells per condition and only proteins detected in all 3 replicates after IFNβ-wt and -ΔS13 stimulation are shown.

Data S3. (separate file)

Source data underlying quantitative figure panels and their statistical analysis.
